# *mir-233* Modulates the Unfolded Protein Response in *C. elegans* during *Pseudomonas aeruginosa* Infection

**DOI:** 10.1371/journal.ppat.1004606

**Published:** 2015-01-08

**Authors:** Li-Li Dai, Jin-Xia Gao, Cheng-Gang Zou, Yi-Cheng Ma, Ke-Qin Zhang

**Affiliations:** Laboratory for Conservation and Utilization of Bio-Resources, Yunnan University, Kunming, Yunnan, China; Massachusetts General Hospital, United States of America

## Abstract

The unfolded protein response (UPR), which is activated by perturbations of the endoplasmic reticulum homeostasis, has been shown to play an important role in innate immunity and inflammation. However, little is known about the molecular mechanisms underlying activation of the UPR during immune responses. Using small RNA deep sequencing and reverse genetic analysis, we show that the microRNA *mir-233* is required for activation of the UPR in *Caenorhabditis elegans* exposed to *Pseudomonas aeruginosa* PA14. *P. aeruginosa* infection up-regulates the expression of *mir-233* in a p38 MAPK-dependent manner. Quantitative proteomic analysis identifies SCA-1, a *C. elegans* homologue of the sarco/endoplasmic reticulum Ca^2+^-ATPase, as a target of *mir-233*. During *P. aeruginosa* PA14 infection, *mir-233* represses the protein levels of SCA-1, which in turn leads to activation of the UPR. Whereas *mir-233* mutants are more sensitive to *P. aeruginosa* infection, knockdown of *sca-1* leads to enhanced resistance to the killing by *P. aeruginosa*. Our study indicates that microRNA-dependent pathways may have an impact on innate immunity by activating the UPR.

## Author Summary

In the model organism *Caenorhabditis elegans*, the IRE1–XBP1 pathway, a major branch of the unfolded protein response (UPR), is required for host defense against pathogens. However, how innate immune responses activate the UPR is not fully understood. In this report, we find that *Pseudomonas aeruginosa* PA14 infection up-regulates the expression of the microRNA *mir-233* in *C. elegans*. The response of *mir-233* to *P. aeruginosa* PA14 infection is dependent on a major pathway of innate immunity, the p38 MAPK signaling cascade. The up-regulation of *mir-233* is functionally important since a mutation in *mir-233* leads to hypersensitivity of the nematode to the killing by *P. aeruginosa* PA14. Furthermore, we demonstrate that *mir-233* contributes to the activation of the UPR by repressing the protein levels of its target SCA-1, a *C. elegans* homologue of the sarco/endoplasmic reticulum Ca^2+^-ATPase. Thus, *mir-233* is an important regulator of the UPR during the innate immune response.

## Introduction

The innate immune system, the first line of defense against microbial infection, is evolutionarily conserved in both vertebrate and invertebrate animals. Activation of the innate immune system upon pathogen infection results in a definitive anti-microbial response to invading microbes. The genetically tractable model organism *Caenorhabditis elegans* has contributed greatly to advancing our understanding of innate immunity in animals [1], [2]. During the last decade, *C. elegans*-based studies have identified a variety of signaling pathways involved in innate immunity, including the p38 mitogen activated protein kinase (MAPK) PMK-1 signaling pathway [2], [3]. The p38 MAPK pathway plays a major role in the immune responses to pathogens, mainly through regulating the expression of secreted antimicrobials, including C-type lectins, ShK toxins, and CUB-like genes [4].

In eukaryotic cells, the endoplasmic reticulum (ER) is a principal site for the folding and maturation of most secreted and transmembrane proteins [5], [6]. Increased load of misfolded proteins that enter the ER can lead to ER stress. To cope with this stress, cells trigger a signal transduction system collectively termed the unfolded protein response (UPR). The UPR is conserved from yeast to human and is an integrated intracellular signaling pathway that links the ER lumen with the cytoplasm and nucleus. Accumulating evidence has revealed that innate immunity is a physiologically relevant source of ER stress in *C. elegans* [7], [8]. The PMK-1 signaling pathway is required for activation of the UPR induced by *Pseudomonas aeruginosa* infection [7] or pore-forming toxins produced by human pathogens, such as *Staphylococcus aureus*, *Streptococcus pyogenes*, and *Aeromonas hydrophilia* [8]. However, the molecular mechanism underlying activation of the UPR by PMK-1 in these processes remains unclear.

MicroRNAs (miRNAs), a class of RNA molecules of 22 nucleotides (nt) long, are pivotal regulators of gene expression in metazoa [9]. In animals, miRNAs mainly target specific mRNAs through imperfect base pairing with the 3′-untranslated region (3′ UTR) of these mRNAs [10]. miRNAs are involved in a wide variety of biological processes, including patterning of the nervous system, inflammation and immunity, cell death and proliferation, and development [11]. Since the first two miRNAs, *lin-4* and *let-7*, were identified as regulators of developmental timing [12], [13], more than 175 of miRNA genes have been confirmed in *C. elegans* [14]. Previous studies have revealed that a reduction in total miRISC activity or mutations in *dcr-1*, *drsh-1* and *alg-1* genes enhances worm resistance to pathogenic bacteria *P. aeruginosa* PA14 or *Bacillus thuringiensis* DB27 [15], [16]. As these genes are required for miRNA processing, these results imply that miRNAs are probably involved in innate immune responses to pathogenic bacteria. Furthermore, Liu et al. [17] have demonstrated that the *mir-84(n4037)* and *mir-241(n4316)* mutants exhibit enhanced resistance, whereas the *mir-48(n4097)* mutant worms exhibit decreased resistance to *P. aeruginosa* infection. Thus, different *let-7* miRNA homologs play distinct roles in innate immune responses to bacterial infection.

To better understand the role of miRNAs in innate immunity, we used RNA deep sequencing to carry out a comprehensive survey of miRNA expression in wild type (WT) animals grown on live *P. aeruginosa* PA14. We screened the up-regulated miRNAs and discovered that *mir-233* was required for resistance to *P. aeruginosa* PA14 infection. Using a proteomic approach, we identified that *sca-1*, which encodes a *C. elegans* homologue of the sarco/endoplasmic reticulum Ca^2+^-ATPase (SERCA) [18], was the target of *mir-233*. Down-regulation of SCA-1 protein levels by *mir-233* resulted in activation of the UPR, which in turn conferred resistance to *P. aeruginosa* PA14 infection. Finally, our data demonstrate that the UPR pathway functions in the intestine, the major site of pathogen exposure.

## Results

### *mir-233* is required for *C. elegans* innate immunity

**Figure 1:**
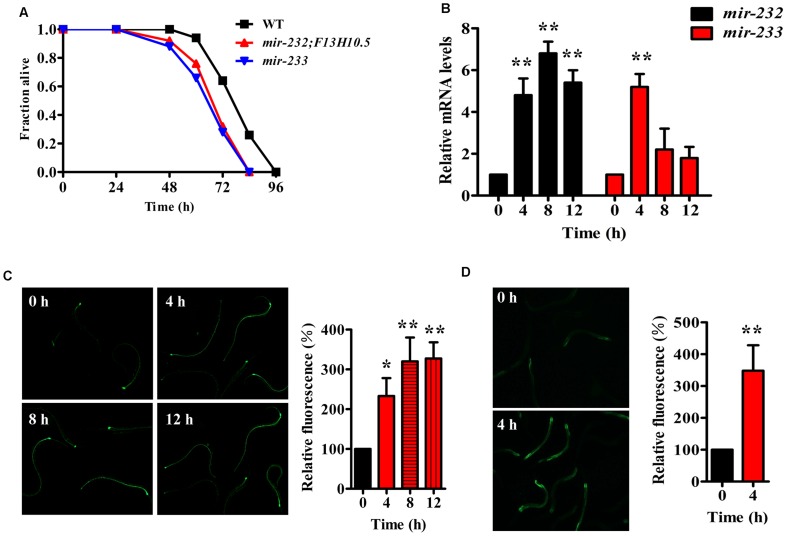
*P. aeruginosa* infection up-regulates the expression of *mir-232* and *mir-233*, which are required for innate immunity. (A) Mutations in *mir-232* and *mir-233* reduced survival of worms exposed to *P. aeruginosa* PA14. The survival graph represents combined results of three independent experiments. *P*<0.01 versus wild type (WT). (B) qRT-PCR analysis of expression of *mir-232* and *mir-233* in WT worms after PA14 infection. The data are expressed as percent of control (the value of “0 hour” time point). **P<0.01 versus worms at 0 h. (C–D) The expression of *mir-232p::gfp* (C) and *mir-233p:: gfp* (D) in WT worms was significantly up-regulated by PA14 infection. The right parts show quantification of GFP levels. *P<0.05; **P<0.01 versus worms at 0 h.

To explore whether miRNAs are involved in innate immunity in *C. elegans*, we determined the miRNA expression profiles in worms exposed to *P. aeruginosa* PA14 using small RNA deep sequencing. We found that 40 miRNAs at 4 h, 68 miRNAs at 8 h, and 64 miRNAs at 12 h post-infection were up-regulated, respectively (S1 Table). We hypothesized that some of the miRNAs up-regulated in response to bacterial infection play a role in *C. elegans* innate immunity. Thus, we focused on the 88 miRNAs and miRNA families that were up-regulated after PA14 infection. To identify individual miRNAs that play prominent roles in innate immunity, we tested 47 available mutant strains of these 88 miRNAs. Whereas mutations in most of the tested miRNAs did not influence the immune phenotype, *mir-232(ndf56);F13H10.5* and *mir-233(n4761)* mutants exhibited enhanced susceptibility to the killing by PA14 (Fig. 1A and S2 Table). Using quantitative RT-PCR (qRT-PCR), we confirmed that the expression of *mir-232* was markedly elevated in worms at 4 h, 8 h, and 12 h after exposure to *P. aeruginosa* PA14, compared with worms grown in the standard laboratory food *Escherichia coli* OP50 (Fig. 1B). Meanwhile, up-regulation of *mir-233* was observed in worms at 4 h post-infection. Furthermore, using transgenic animals that express *mir-232p::gfp* or *mir-233p::gfp*, we observed that PA14 infection significantly increased expression of *mir-232p::gfp* (Fig. 1C) and *mir-233p::gfp* (Fig. 1D).

The *mir-233(n4761)* mutant is a deletion that removed not only *mir-233*, but also the gene *W03G11.4*. However, knockdown of *W03G11.4* by RNAi had no impact on survival of WT worms after *P. aeruginosa* PA14 infection (S1A–S1C Fig.). Thus, the immune-deficient phenotype of the *mir-233(n4761)* mutant was not due to the removal of *W03G11.4*. Likewise, the *mir-232(ndf56);F13H10.5* mutant animals also have two mutations. We found that knockdown of *F13H10.5* by RNAi led to enhanced resistance to PA14 infection (S1D Fig.). These results suggest that the mutations in *mir-232(ndf56)* and *F13H10.5* display a mixed effect on innate immunity. Thus, we focused on the role for *mir-233* in innate immunity. In addition to its defect in a response to *P. aeruginosa* PA14 infection, the *mir-233(n4761)* worms were more sensitive to killings by the Gram-positive bacteria *S. aureus* ATCC 25923, *Enterococcus faecalis* ATCC 29212, and the Gram-negative bacterium *Salmonella typhimurium* 468 than WT animals (S2A–S2C Fig.). Furthermore, we found that the *mir-233(n4761)* mutant exhibited a comparable lifespan to WT worms (S3 Fig.), suggesting that the effect of *mir-233* on the defense against *P. aeruginosa* is not due to changes in aging.

### SCA-1 is a target of *mir-233*

**Figure 2:**
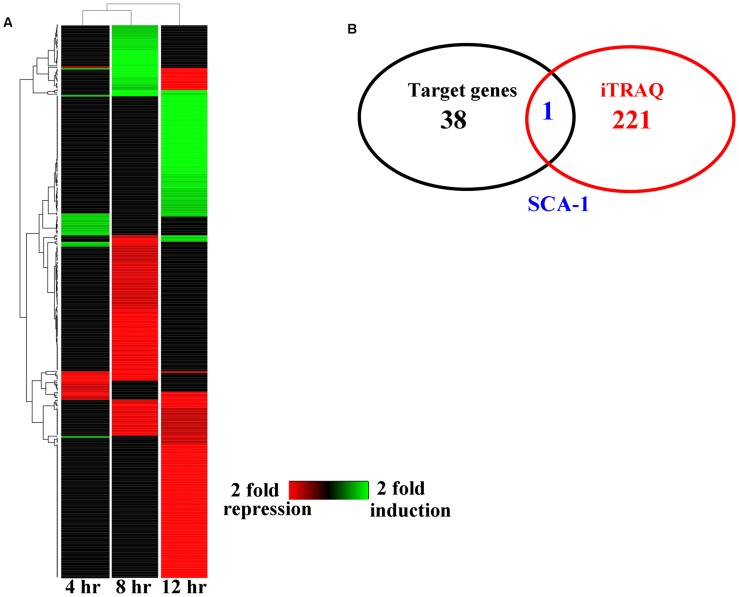
The changes of protein levels in worms after *P. aeruginosa* infection. (A) Heat maps show significantly up- and down-regulated proteins after *P. aeruginosa* PA14 infection. Protein expression profiles were clustered hierarchically. (B) A Venn diagram comparing the overlap in target genes of *mir-233* and proteins down-regulated by *P. aeruginosa* PA14 infection.

Animal miRNAs usually bind to their target mRNAs, thus resulting in down-regulation of protein translation. It is reasoned that in response to bacterial infection, *mir-233* functions to silence genes that have a negative impact on *C. elegans* innate immunity. Three different miRNA target prediction algorithms (MICRORNA, TargetScan, and DIANA-microT) suggested 38 putative target genes of *mir-233* (S4 Fig.). To confirm the target genes of *mir-233* that are involved in innate immunity, we used isobaric tags for relative and absolute quantitation (iTRAQ)-based quantitative proteomic approach to analyze the early changes in protein levels in worms exposed to *P. aeruginosa* PA14. As a commonly used quantitative proteomics method, iTRAQ allows for simultaneous comparative quantification of multiple samples within a single run, leading to a reduction of the experimental errors produced from individual experiments [19]. We found that the expression of 51, 45, and 85 proteins were up-regulated, while 19, 115, and 133 proteins were down-regulated at 4 h, 8 h, and 12 h post-infection, respectively (Fig. 2A and S3, S4, S5 Tables). However, when comparing our protein data set to two microarray data sets from the studies published by Shapira et al [20] and Troemel et al [4], we found very little overlap between our protein data set and the two microarray data sets. These results suggest that these gene expression changes are not reflected in our proteomic analysis. Many factors such as posttranscriptional regulation, variable rates of protein turnover, and post-translational modification, may account for the observed discordance between transcript and protein levels [21].

**Figure 3:**
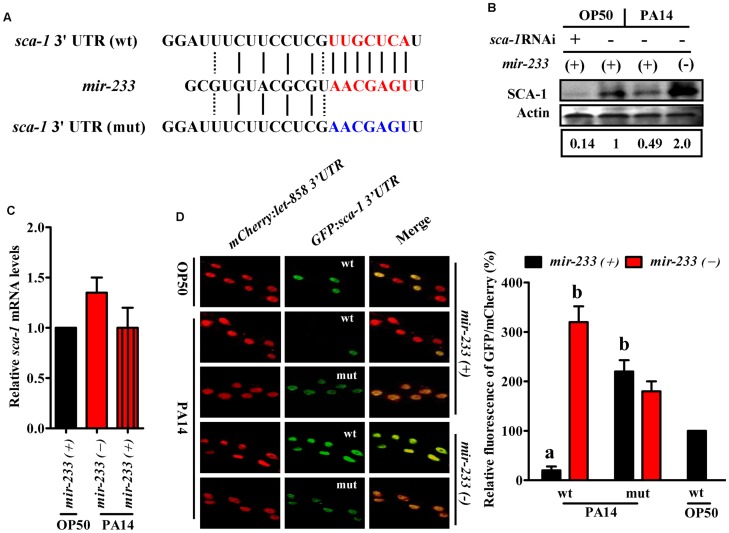
*sca-1* is a target gene of *mir-233*. (A) Complementarity between the 3′ UTR of *sca-1* gene and *mir-233*. *mir-233* is presented in the form of DNA from 3′ to 5′ end. WT seeds are marked in red, mutated seeds in blue, Watson-Crick base pairing with a straight line and U-G wobbles with a dotted line. For constructing of *sca-1*-3′UTR (mut) reporter, the putative *mir-233* binding site was replaced with an oligonucleotide containing the exact complementary sequence of *mir-233*. (B) The protein levels of SCA-1 were determined by Western blotting at 8 h after PA14 infection. The blot is typical of three independent experiments. *P*<0.05, *mir-233(+)* (wild type worms)+PA14 versus *mir-233(+)*+OP50; *P*<0.01, *mir-233(-)* (*mir-233(n4761)* mutant worms)+PA14 versus *mir-233(+)*+PA14; *mir-233(+)*+OP50+*sca-1* RNAi versus *mir-233(+)*+OP50. (C) Deletion of *mir-233* did not affect the mRNA levels of *sca-1*. (D) Fluorescence images of the *sca-1*-3′ UTR GFP reporter in worms grown on *E. coli* OP50 or *P. aeruginosa* PA14. Mutagenesis of the putative binding site for *mir-233* in the *sca-1*-3′UTR led to derepression of the GFP expression in *mir-233(+)* worms after PA14 infection. Meanwhile, the GFP expression was significantly higher in *mir-233(−)* worms than that in *mir-233(+)* worms after PA14 infection. The lower panel shows quantification of GFP/mCherry ratio. The data are expressed as percent of control (the ‘wt’ construct on OP50). ^a^ *P*<0.01 versus *mir-233(+)*+wt on OP50; ^b^ *P*<0.01 versus *mir-233(+)*+wt on PA14.

We found only one gene (*sca-1*) overlapped between the down-regulated-protein encoding genes and the target genes of *mir-233* (Fig. 2B). The protein levels of SCA-1 were markedly reduced at 8 h post-infection (Fig. 2A and S4 Table). miRNA target prediction algorithms revealed that the *sca-1* gene contained one putative binding site for *mir-233* in the 3′UTR region (Fig. 3A). SCA-1 is the *C. elegans* homologue of the mammalian SERCAs, which shows about 70% amino acid sequence identity and 80% similarity to the three human SERCA proteins [18]. Using Western blotting, we found that the protein levels of SCA-1 were decreased in WT worms at 8 h after *P. aeruginosa* PA14 infection (Fig. 3B). However, the protein levels of SCA-1 in the *mir-233(n4761)* mutant were significantly higher than those in WT worms after *P. aeruginosa* PA14 infection (Fig. 3B). In contrast, the mRNA levels of *sca-1* in WT and the *mir-233(n4761)* animals were similar after PA14 infection (Fig. 3C), indicating that transcriptional regulation is unlikely to be involved in the reduced SCA-1 protein levels. To address whether *mir-233* regulated the protein levels of SCA-1 through 3′UTR, we constructed a 4×NLS::GFP vector driven by the *rpl-28* promoter, which contained the 3′UTR of *sca-1* (*Prpl-28::gfp:sca-1* 3′UTR) [22]. Meanwhile, a *sca-1*-3′UTR mutant reporter construct was generated by replacing the putative *mir-233* binding site with an oligonucleotide containing the exact identical sequence of *mir-233*. Specially, an *Prpl-28::histone-24::mCherry:let-858* 3′UTR construct that drives constitutive and mCherry expression was used as an internal control. We found that the GFP expression was markedly reduced in WT worms at 8 h after *P. aeruginosa* PA14 infection (Fig. 3D). However, mutagenesis of the putative binding site for *mir-233* in the *sca-1*-3′UTR abolished the inhibition of the GFP expression in WT worms. Furthermore, we found that the GFP expression was much higher in the *mir-233(n4761)* mutant than that in WT worms after PA14 infection (Fig. 3D). These results suggest that *mir-233* suppresses the protein levels of SCA-1 through binding to its 3′UTR and inhibiting its translation after PA14 infection.

### SCA-1 is a negative regulator of innate immunity in *C. elegans*

**Figure 4:**
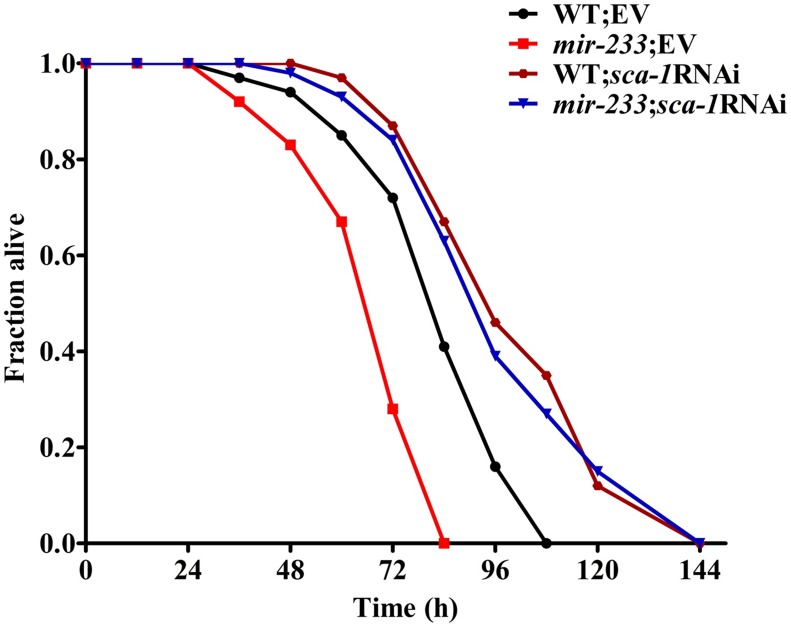
*sca-1* RNAi leads to enhanced resistance to *P. aeruginosa* infection. The survival graph represents combined results of three independent experiments. *P*<0.01 versus wild type worms (WT)+empty vector (EV).

A mutation in *sca-1(mca-4)* has shown to result in embryonic and larval lethality [18], [23]. To test the role of *sca-1* in pathogen susceptibility, we reduced the expression of the gene by RNAi. However, as demonstrated previously, the majority of *sca-1*(RNAi) worms died or were arrested at the L1 stage [18], [23], [24]. To overcome this problem, we diluted the *sca-1* RNAi bacteria with an empty vector control at a ratio of 1 to 4. This dilution modified the severity of the phenotype enough to enable development. qRT-PCR analysis demonstrated that knockdown of *sca-1* by RNAi in 1/4 dilution reduced approximately 50% of s*ca-1* mRNA levels (S5 Fig.). The 1/4 *sca-1* RNAi exhibited enhanced resistance to *P. aeruginosa* PA14 infection in WT worms, compared to those subjected to the vector control RNAi (Fig. 4).

Next, we determined whether up-regulated expression of SCA-1 protein contributed to the immune-deficient phenotype of the *mir-233(n4761)* mutant. The *mir-233(n4761)* mutant subjected to *sca-1* RNAi showed markedly increased survival relative to the *mir-233(n4761)* mutant exposed to the empty vector, to a degree that was comparable to the survival of WT worms subjected to *sca-1* RNAi (Fig. 4). These results suggest that *mir-233* is involved in innate immunity by suppressing SCA-1 expression.

### *mir-233* initiates the UPR by suppressing SCA-1

**Figure 5:**
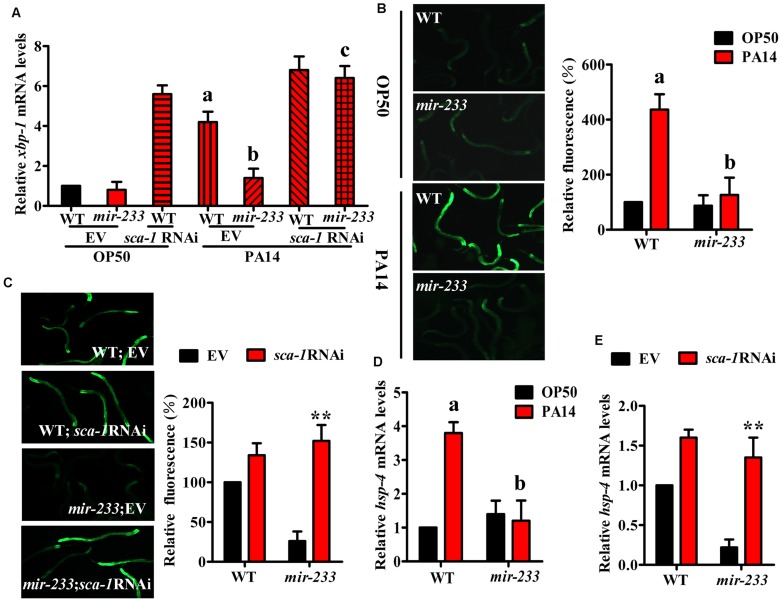
*mir-233* mediates the XBP-1-dependent UPR induced by *P. aeruginosa* infection. (A) qRT–PCR analysis of spliced *xbp-1* mRNA levels at 8 h after *P. aeruginosa* PA14 infection. ^a^ *P*<0.01 versus wild type (WT) worms+EV on *E. coli* OP50; ^b^ *P*<0.05 versus WT+EV on PA14; ^c^ *P*<0.01 versus *mir-233(n4761)* on PA14. (B) Fluorescence images of WT and *mir-233(n4761)* animals carrying the *Phsp-4::gfp* transgene at 8 h after PA14 infection. The data are expressed as percent of control (WT on OP50). ^a^ *P*<0.01 versus WT on OP50; ^b^ *P*<0.05 versus WT on PA14. (C) *sca-1* RNAi restored the expression of *Phsp-4::gfp* in the *mir-233(n4761)* worms grown on *P. aeruginosa* PA14. **P<0.01 versus *mir-233*+empty vector (EV). (D–E) qRT-PCR analysis of *hsp4* mRNA levels after PA14 infection. (D) ^a^ *P*<0.01 versus WT on OP50; ^b^ *P*<0.05 versus WT on PA14. (E) **P<0.01 versus *mir-233*+EV.

A previous study has demonstrated that *P. aeruginosa* PA14 infection activates the UPR [7]. The UPR is comprised of three branches, the ribonuclease inositol-requiring protein–1 (IRE-1), the PERK kinase homologue PEK-1, and the transcription factor ATF6 [5], [6]. In *C. elegans*, the IRE-1-XBP-1 pathway is required for resistance to *P. aeruginosa* infection or to the treatment of pore-forming toxins [7], [8]. IRE-1 splices an intron from XBP1 mRNA, producing the activated ‘spliced form’ of XBP-1 (XBP-1s) [5], [6]. As *sca-1* RNAi leads to the UPR [25], we tested the role of *mir-233* in the UPR. First, the levels of *xbp-1s* transcript were increased approximately 4-fold in WT worms, but not in the *mir-233(n4761)* mutant, at 8 h after *P. aeruginosa* PA14 infection (Fig. 5A). However, *sca-1* RNAi restored the expression of *xbp-1s* in the *mir-233(n4761)* mutant exposed to PA14. The induction of BiP/GRP78, a molecular chaperone, reflects activation of the IRE-1-XBP-1 branch of the UPR [5], [6]. In *C. elegans*, *hsp-4* gene encodes a homologue of mammalian BiP/GRP78, which is a target of XBP-1. Second, we detected the UPR activation using transgenic worms carrying *Phsp-4::gfp* [26], and found that *P. aeruginosa* PA14 infection induced the expression of *Phsp-4::gfp* in WT worms, but not in the *mir-233(n4761)* mutant (Fig. 5B). Furthermore, the expression of *Phsp-4::gfp* in the *mir-233(n4761)* mutant was significantly restored by *sca-1* RNAi after PA14 infection (Fig. 5C). Similar results were obtained using qRT-PCR analysis for the mRNA levels of *hsp-4* (Fig. 5D and 5E). Taken together, these results indicate that *mir-233* is critically involved in silencing the *sca-1* transcript to activate the IRE-1/XBP-1 pathway.

A previous study has suggested that the effect of the IRE-1/XBP-1 signaling on worm survival upon *P. aeruginosa* infection is due to increased tolerance and not increased resistance [7]. Here we tested *P. aeruginosa* accumulation in the intestine of the *mir-233(n4761)* mutant or worms subjected to *sca-1* RNAi. We found that, like a mutation in *xbp-1(zc12)*, the mutation in *mir-233* or knockdown of *sca-1* had no impact on the colony forming units (CFU) of PA14 in worms (S6A Fig.). Moreover, the accumulation of *P. aeruginosa* expressing GFP in the *mir-233(n4761)* or *sca-1*(RNAi) worms was comparable to that observed for WT worms with empty vector (S6B Fig.). In contrast, a mutation in *pmk-1(km25)*, a conserved p38 MAPK pathway that plays a crucial role in innate immunity, resulted in a significant increase in accumulation of *P. aeruginosa* [7] (S6A–S6B Fig.). These results implicate that innate immune pathways may play distinct roles in the pathogenesis of infectious diseases.

### PMK-1-mediated activation of the UPR through the *mir-233*/SCA-1 pathway

**Figure 6:**
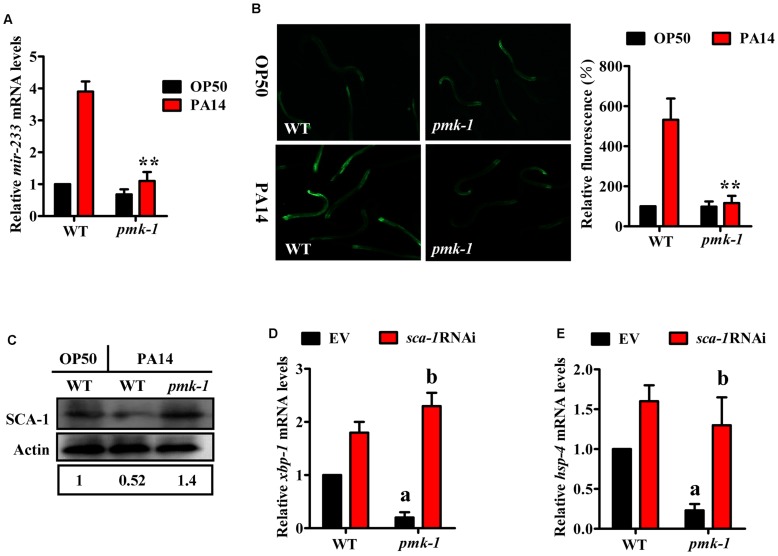
The *mir-233*/SCA-1 signaling functions as a downstream effector of p38 MAPK in the activation of the UPR. (A) qRT-PCR analysis of *mir-233* expression in wild type (WT) and *pmk-1(km25)* worms at 4 h after *P. aeruginosa* PA14 infection. **P<0.01 versus WT on PA14. (B) The expression of *mir-233p::gfp* was significantly down-regulated by *pmk-1* RNAi at 4 h after *P. aeruginosa* PA14 infection. The right parts show quantification of GFP levels. The data are expressed as percent of control (WT). **P<0.01 versus WT on PA14. (C) The protein levels of SCA-1 were determined by Western blotting after *P. aeruginosa* PA14 infection for 8 h. The blot is typical of three independent experiments. *P*<0.05, WT+PA14 versus WT+OP50; *P*<0.01, *pmk-1(km25)*+PA14 versus WT+PA14. (D–E) The expressions of *xbp-1s* (D) and *hsp-4* (E) were determined by qRT-PCR at 8 h after PA14 infection. (D) ^a^ *P*<0.01 versus WT+empty vector (EV); ^b^ *P*<0.01 versus *pmk-1(km25)*+EV. (E) ^a^ *P*<0.01 versus WT+EV; ^b^ *P*<0.05 versus *pmk-1(km25)*+EV.

A previous study has shown that activation of the UPR in response to *P. aeruginosa* infection or to the pore-forming toxins produced by many human pathogens is mediated by the p38 MAPK orthologue PMK-1 [3], [8]. These results raised the possibility that PMK-1 could regulate the UPR through the *mir-233*/SCA-1 pathway. To test this hypothesis, we first examined the effect of PMK-1 on *mir-233* expression. qRT-PCR analysis indicated that a mutation in *pmk-1(km25)* led to a significant decrease in both the levels of *mir-233* and the expression of *mir-233p::gfp* at 4 h after PA14 infection (Fig. 6A and 6B). Second, the protein levels of SCA-1 in the *pmk-1(km25)* mutant were markedly higher than those in WT worms during PA14 infection (Fig. 6C). Finally, the expressions of *xbp-1s* and *hsp-4* were reduced in the *pmk-1(km25)* mutant, compared with WT worms after PA14 infection (Fig. 6D and 6E), consistent with the observation reported by Richardson et al [7]. However, *sca-1* RNAi restored the expressions of *xbp-1s* and *hsp-4* in the *pmk-1(km25)* mutant to the same levels as WT worms (Fig. 6D and 6E). These results suggest that activation of the IRE-1/XBP-1 signaling by PMK-1 is through the *mir-233*/SCA-1 pathway.

It has been shown that the transcription factor ATF-7 functions as a regulator of PMK-1-mediated innate immunity via repression of immune gene expression [27], raising a possibility that the PMK-1-induced expression of *mir-233* is mediated by ATF-7. To test this hypothesis, we tested the role of ATF-7 on *mir-233* expression. However, we found that the expression of *mir-233* in the *atf-7(gk715)* mutant was comparable to that in WT worms after PA14 infection (S7A Fig.). Meanwhile, *atf-7* RNAi did not influence the expression of *mir-233p::gfp* in either WT or *pmk-1(km25)* worms after PA14 infection (S7B Fig.). Thus, ATF-7 is not involved in the induction of *mir-233* mediated by PMK-1.

### The UPR in the intestine is involved in resistance to *P. aeruginosa* infection

The IRE-1-XBP-1 signaling has shown to be required for resistance to *P. aeruginosa* infection during larval development [7]. We found that, like WT worms, all the *sca-1*(RNAi) worm eggs grown on plates with *P. aeruginosa* PA14 as the only food source could develop to the fourth larval stage (L4), comparable to their development on *E. coli* OP50 (S8 Fig.). In contrast, unlike WT or *sca-1*(RNAi) worms, the *mir-233(n4761)* mutant on *P. aeruginosa* PA14 plates exhibited severely attenuated larval development, and approximately 60% larva could not reach the L4 stage by 72 h (S8 Fig.).

**Figure 7:**
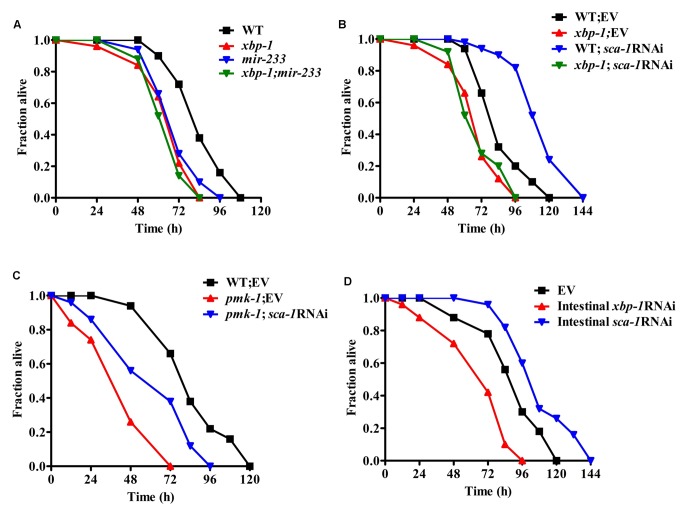
The SCA-1/XBP-1 pathway in the intestine functions as a regulator of innate immunity mediated by PMK-1. (A) Both *xbp-1(zc12)* and *mir-233(n4761)* mutants were more sensitive than wild type (WT) worms to the killing by *P. aeruginosa* PA14. *P*<0.01 versus WT. However, the mutation in *mir-233(n4761)* did not enhance susceptibility of the *xbp-1(zc12)* mutant to PA14 infection. (B) Although knockdown of *sca-1* by RNAi promoted the survival of worms to PA14 infection, the survival of *xbp-1(zc12)*; *sca-1* RNAi worms was comparable to that of the *xbp-1(zc12)* worms after PA14 infection. *P*<0.01 versus WT+empty vector (EV). (C) The survival of the *pmk-1(km25)* mutant was partially restored by knockdown of *sca-1*. *P*<0.001, *pmk-1(km25)* versus WT; *P*<0.01, *pmk-1(km25)*; *sca-1* RNAi versus *pmk-1(km25)*+EV. (D) Intestinal-specific RNAi of *sca-1* enhanced resistance, whereas intestinal-specific RNAi of *xbp-1* led to enhanced sensitivity to PA14 infection. *P*<0.01 versus control (EV). All the survival graphs represent combined results of three independent experiments.

Next, we asked if XBP-1 was required for resistance to *P. aeruginosa* PA14 in adult worms. We found that the *xbp-1(zc12)* mutant exhibited enhanced sensitivity of young adult worms to the killing by *P. aeruginosa* PA14 (Fig. 7A). It should be noted that the lifespan of the *xbp-1(zc12)* mutant was similar to that of WT worms (S9 Fig.), suggesting that the impact of XBP-1 on the defense against *P. aeruginosa* is not due to changes in aging. It has been reported that PEK-1, a branch of the UPR that is distinct from the IRE-1-XBP-1 pathway, is important for the worm resistance to *P. aeruginosa* infection under lower temperatures [28]. However, we found that the survival of the *pek-1(ok275)* mutant was comparable to that of WT animals after PA14 infection at standard temperature (25°C) (S10 Fig.).

The survival of the *xbp-1(zc12);mir-233(n4761)* double mutants was comparable to that of the *xbp-1(zc12)* mutant worms after PA14 infection (Fig. 7A). Although *sca-1*(RNAi) worms were more resistant to PA14 infection than WT worms, the survival of *xbp-1(zc12); sca-1* RNAi was comparable to that of the *xbp-1(zc12)* mutant after *P. aeruginosa* PA14 infection, suggesting that the immune-resistant phenotype of *sca-1*(RNAi) worms is XBP-1-dependent (Fig. 7B). Unlike the *mir-233(n4761)* mutant, the immune-deficient phenotype of the *pmk-1(km25)* mutant was only partially rescued by knockdown of *sca-1* (Fig. 7C). These results implicate that the UPR signaling is one of the downstream effectors in PMK-1-mediated innate immunity. Indeed, the survival of the *xbp-1;pmk-1* double mutants was comparable to that of the *pmk-1(km25)* mutant (S11 Fig.).

We noticed that *mir-233* was highly expressed in the intestine of *C. elegans* (S12 Fig.). We found that intestinal-specific *sca-1* RNAi resulted in enhanced resistance to PA14 infection (Fig. 7D), whereas epidermal- and muscular-specific *sca-1* RNAi did not affect the survival of worms (S13A–S13B Fig.). Likewise, intestinal-specific RNAi of *xbp-1* enhanced sensitivity to PA14 infection (Fig. 7D), whereas epidermal- and muscular specific RNAi of *xbp-1* failed to affect the survival of worms (S14A–S14B Fig.). These data suggest that the UPR in the intestine probably is required for innate immunity.

## Discussion

The accumulation of unfolded proteins beyond the levels that ER can cope with leads to ER stress, which in turn activates ER stress signaling, the UPR [6]. Many of the molecular chaperones within the ER, such as GRP78, calnexin and calreticulin, are Ca^2+^ binding proteins [29]. Thus, the luminal Ca^2+^ is crucial for proper folding and maturation of proteins in the ER [30]. As a Ca^2+^-ATPase, the SERCA pump is responsible for transferring Ca^2+^ from the cytosol to the ER lumen via ATP hydrolysis [31]. In mammalian cells, inhibition of SERCA activity or down-regulation of SERCA expression is among the main mechanisms that evoke the UPR under a variety of pathophysiological conditions [30], [32]. For example, the oxidized LDL can induce oxidative stress in endothelial cells, which in turn inhibits SERCA by enhancing oxidation of the Cys674 residue of these ATPases [32]. Disturbed ER calcium stores leads to the UPR. In *C. elegans*, the UPR is induced in response to *P. aeruginosa* infection or to pore-forming toxins produced by a variety of human pathogens, suggesting that the innate immune responses represent a physiologically relevant inducer of the UPR [7], [8]. Our study reveals two novel regulatory components of the UPR: *mir-233* and SCA-1. During *P. aeruginosa* infection, *mir-233* negatively regulates the protein expression of SCA-1 through direct base pairing to the 3′ UTR of its mRNA. Suppression of SCA-1, in turn, promotes the activation of the UPR, which confers resistance to the pathogen.

In the current study, *mir-233* was identified by screening 47 up-regulated miRNAs for susceptibility to *P. aeruginosa* infection. The *mir-233(n4761)* mutant animals were found to be susceptible to several other bacterial pathogens. These results suggest that *mir-233* is required for immune responses to pathogenic bacteria in general. As demonstrated previously [33], [34], the *mir-233(n4761)* mutant animals showed normal phenotypes including locomotion, egg laying, pumping, and defecation. In addition, we found that a mutation in *mir-233(n4761)* did not influence the lifespan of worms. Thus, the pathogen killing assays reflect the effect of *mir-233* on host defense against attacking pathogens, not *mir-233*-dependent changes in aging.

Previously, Horvitz and colleagues have revealed that a single mutation of the majority of miRNAs does not lead to obvious developmental or growth defects of worms [33], [34]. These observations lead them to hypothesize that there is significant functional redundancy among miRNAs. Our results reinforce the idea that most miRNAs act redundantly with other miRNAs or other pathways. Although 47 miRNA genes were up-regulated after *P. aeruginosa* PA14 infection, single mutations in most of these miRNAs did not influence innate immune responses to PA14 infection. However, we could not exclude the role of these miRNAs in innate immune responses to other microbes. It is possible that innate immunity is regulated by multiple miRNAs and a large number of target genes, which together consist of the miRNA-target network. Thus, a single mutation in these miRNAs does not de-repress the expression of immune-related genes as the other miRNAs that might regulate in parallel the target genes.

Our results demonstrate that *mir-233* down-regulates the protein levels of its target gene SCA-1 during *P. aeruginosa* PA14 infection. SCA-1 is identified by iTRAQ analysis and miRNA target prediction algorithms. It should be noted that there was little overlap between the expressions of proteins at each of the time-points. The data from the proteomic analysis probably varied greatly, contributing to the statistically no significant difference between the expressions of proteins at each of the time-points. SCA-1 is the only *C. elegans* homolog of mammalian SERCA [18], which is required for the maintenance of intracellular Ca^2+^ homeostasis. In *C. elegans*, down-regulation of *sca-1* by RNAi leads to Ca^2+^ depletion, thus inducing activation of the UPR [25]. Thus, these results imply a role for *mir-233* in regulating the UPR during the immune response. Indeed, an increase in expression of *xbp-1s* and *Phsp-4::gfp* in response to PA14 infection are only observed in WT worms, but not in the *mir-233(n4761)* mutant. It has been shown that innate immunity constitutes a physiologically relevant source of ER stress in *C. elegans* [7], [8]. Although the molecular mechanism by which innate immunity-induced UPR is not clear, PMK-1, which plays a principal role in *C. elegans* defense against pathogens, is involved in the activation of the UPR. Our data demonstrate that PMK-1 mediates up-regulation of *mir-233* expression after *P. aeruginosa* PA14 infection. In contrast, a mutation in *pmk-1* leads to an increase in the protein expression of SCA-1. Meanwhile, knockdown of *sca-1* by RNAi restores the induction of *xbp-1s* in the *pmk-1(km25)* mutant. Thus, PMK-1 promotes the activation of the UPR by regulating the *mir-233*/SCA-1 signaling during the immune response.

Of the three canonical braches of the UPR, the IRE-1-XBP-1 signaling is involved in resistance to *P. aeruginosa* and to pore-forming toxins in *C. elegans* [7], [8]. Our results demonstrate that knockdown of *sca-1* by RNAi enhances immune resistance to *P. aeruginosa* infection in adult WT worms, but not in the *xbp-1(zc12)* mutant. Moreover, intestinal-specific knockdown of *xbp-1* leads to enhanced susceptibility to PA14 infection. These results indicate that *mir-233*-mediated activation of the UPR in the intestine, which is in direct contact with pathogens, confers defense against *P. aeruginosa* infection.

The protective role of the UPR in innate immunity is not fully understood. During larval development, *P. aeruginosa* infection induces the PMK-1-dependent innate immune response, which in turn causes a disruption of ER homeostasis in the absence of the functional IRE-1–XBP-1 pathway [7]. Thus, Richardson et al [7] suggest that the principal mechanism by which XBP-1 promotes survival of worms during *P. aeruginosa* infection is to alleviate the detrimental effect induced by the immune response. These authors demonstrate that the UPR is also induced in response to collateral damage caused by activation of the p38 pathway, which is not associated with bacterial infection. These results, however, raises the possibility that the UPR regulated by *mir-233* contributes to a more general stress resistance, but not innate immunity *per se*. Indeed, the UPR, which is activated by hypoxia, is required for protecting worms against hypoxic injury [35], [36]. As organisms are faced with a variety of stresses that results in protein misfolding and aggregation, the maintenance of the ER proteome may be a common mechanism to coordinate stress resistance.

In summary, we report that *mir-233* is up-regulated by PMK-1 during *P. aeruginosa* PA14 infection. *mir-233* down-regulates the protein expression of its target SCA-1, resulting in activation of the UPR. The UPR in turn confers *C. elegans* defense against *P. aeruginosa* PA14 infection. Thus, *mir-233* is a key miRNA that modulates the UPR during the immune response.

## Materials and Methods

### Nematode strains

Mutated and transgenic strains used in this study include: *xbp-1(zc12)*, *pmk-1(km25)*, *ZcIs[hsp-4::gfp]*, *wwEx33[mir-232p::gfp+unc-119(+)]*, *NR*222 *(rde-1(ne219); kzIs9[pKK1260(plin-12::nls::gfp)*, *pKK1253(plin-26::rde-1)*, *rol-6])*; *NR350 (rde-1(ne219); kzIs20[pDM#715(phlh-1::rde-1)*, *pek-1(ok275)*, and *atf-7(gk715)* were kindly provided by the Caenorhabditis Genetics Center (CGC). The strain for intestinal-specific RNAi *(sid-1(qt9); Is[vha-6pr::sid-1]; Is[sur-5pr:: gfp::nls])* were kindly provided by Dr. Gary Ruvkun (Massachusetts General Hospital, Harvard Medical School). miRNA mutants (from CGC) used in this study were listed in S6 Table. Mutants and transgenic strains were backcrossed three times into the N2 strain used in the laboratory. All strains were maintained on nematode growth media (NGM) and fed with *E. coli* OP50.

### Infection with bacteria

Standard conditions were used for *C. elegans* growth at 20°C [37]. Synchronized populations of worms were cultivated at 20°C until the young adult stage. For all pathogen assays, 75 µg/ml of fivefluoro-2′-deoxyuridine (FUdR) was added to the assay plates to abolish the growth of progeny. *P. aeruginosa* PA14 (a gift from Dr. K Zhu, Institute of Microbiology, CAS) or *E. faecalis* ATCC 29212, or *S. typhimurium* 468, or *S. aureus* ATCC 25923 (gifts from Dr. WH Lee, Kunming Institute of Zoology, CAS) was grown in different medium [4], [38], [39], then seeded on slow-killing plates, which contained modified NGM (0.35% instead of 0.25% peptone) as described [4], [39]. Three plates of about 50–60 animals per plate were tested per assay and all experiments were performed three times independently at 25°C.

### Small RNA deep sequencing

After infected with *P. aeruginosa* PA14 infection for 4 h, 8 h, and 12 h, worms were washed with M9 buffer for several times in order to remove bacteria. Then, worms were collected and RNAs were extracted using the mirVana miRNA Isolation Kit (Ambion), according to the manufacturer's instructions. Approximately 20 µg of RNAs per sample were submitted to Beijing Genomics Institute (BGI)-Shenzhen (Shenzhen, China) for sequencing. In brief, the sequencing was performed as follows: RNAs corresponding to 15–30 nt in size were purified by polyacrylamide gel electrophoresis (PAGE), and ligated with adapters to the 5′ and 3′ termini of the RNA. Then the RNAs were amplified by RT-PCR. cDNA libraries were sequenced using an Illumina Genome Analyzer. Illumina data can be found in the Gene Expression Omnibus (GEO) of NCBI under the accession number GSE17095742.

### RNA interference

The clones of genes for RNAi were from the Ahringer library [40]. RNAi feeding experiments were performed on synchronized L1 larvae at 20°C for 40 h.

### Development assays

The development assays were performed as described previously [7]. Strains (*mir-233(n4761)* mutant, *sca-1*(RNAi), and *mir-233(n4761)*; *sca-1*(RNAi) worms) were egg laid on plates of PA14 (at least 80 eggs for each strain), and the fraction of worms growing to at least the L4 larval stage between the plates was averaged. Development was monitored daily for three days for experiments conducted at 25°C.

### Construction of *W03G11.4* RNAi

To generate a clone directed against *W03G11.4*, a 1420 bp fragment was amplified from genomic DNA by PCR using the primers 5′-GAC ATT ATG GTT GCT TCG-3′ (F) and 5′-GAG ATG CTG AGG TGA GAG-3′ (R). The fragment was TA-cloned into a PstI and KpnI-linearized L4440 feeding vector, as in the RNAi library [41].

### Construction of transgenic strains

The vector *mir-233p::gfp* was generated by subcloning a 2.5 kb promoter fragment of *mir-233* into an expression vector (pPD95.75). The construct was co-injected with the marker plasmid pRF4 containing *rol-6(su1006)* into gonads of WT worms by standard techniques [42], respectively. The transgenic worms were confirmed prior to assay. The expression of GFP was observed under a Zeiss Axioskop 2 plus fluorescence microscope (Carl Zeiss, Jena, Germany). Three plates of about 30 animals per plate were tested per assay and all experiments were performed three times independently.

### Western blotting

After worms were homogenized in liquid nitrogen, the homogenate was lysed on ice for 30 min in lysis buffer (BioTeKe, Beijing, China). The proteins of lysates (50 µg per well) were separated on a 7% SDS-polyacrylamide gel. Proteins were then transferred to immobilon-PSQ transfer PVDF membrane (Millipore, Bedford, MA). Primary antibodies were anti-ATP2A2/SERCA2 antibodies (1∶1000 dilution; Cell Signaling, Beverly, MA), and anti-actin antibodies (1∶1000 dilution; Santa Cruz Biotech., Santa Cruz, CA). The secondary antibody was a peroxidase-coupled anti-rabbit IgG (1∶8000 dilution; Santa Cruz Biotech.). The membrane was exposed to Kodak X-OMAT film (Kodak, Xiamen, China), and the film was developed.

### 3′UTR reporters and microscopy

The ppD129.57 plasmid (a gift from Dr. Min Han, University of Colorado, Boulder, USA), which contains *rpl-28* promoter:*4NLS::gfp:let-858* 3′UTR, was used as the vector for 3′UTR reporter constructs. The 3′UTRs of *sca-1* were PCR amplified from genomic DNA and cloned into ppD129.57 to replace the *let-858* 3′UTR and make a *sca-1*-3′UTR (wt) reporter construct. A *sca-1*-3′UTR (mut) reporter construct was generated by replacing the putative *mir-233* binding site with an oligonucleotide containing the exact complementary sequence of *mir-233*. The 3′UTR reporter constructs and the mCherry internal control plasmid (also a gift from Dr. M. Han) were coinjected into the gonad of WT and *mir-233(n4761)* worms following standard protocols. The transgenic worms were confirmed prior to assay. The expression of GFP and mCherry was monitored using a Zeiss Axioskop 2 plus fluorescence microscope.

### Fluorescence analysis

ImageJ program (NIH) was used to quantify the fluorescence intensity of GFP or mCherry fluorescence. Equal regions of the worm were selected, and the intensities of fluorescence within the selected regions were measured with a standard size of 516×564 pixels. The mean pixelintensity (total pixel intensity/area) for each frame was then calculated. We expressed the fluorescence signal (F) as a ratio with the baseline fluorescence (F0). More than 30 worms per plate were observed to calculate the mean fluorescence intensity. Three plates were tested per assay and all experiments were performed three times independently.

### Quantitative real-time PCR and detection of *xbp-1* splicing

Total RNA was isolated from worms with the mirVana miRNA Isolation Kit (Ambion). Random-primed cDNAs were generated by reverse transcription of the total RNA samples with SuperScript II (Invitrogen). A real time-PCR analysis was conducted using SYBR Premix-Ex TagTM (Takara, Dalian, China) on an Applied Biosystems Prism 7000 Sequence Detection System (Applied Biosystems, Foster City, CA). *act-1* was used for an internal control. The primers used for PCR were as follows: *hsp-4*: 5′- TCA ATG ACG ACG ACA CGC -3′ (F), 5′- CTC CAG AAC TTC GAG ACG G -3′ (R); *xbp-1* splicing: 5′-ACC GTC TGC TCC TTC CTC AATG-3′ (F), 5′- ACC GTC TGC TCC TTC CTC AAT G-3′ (R); *act-1*: 5′-GGG CGA AGA AGG AAA TGG TC-3′ (F), 5′- CAG GTG GCG TAG GTG GAG AA -3′ (R).

### iTRAQ-labeling proteomic analysis

After infected with *P. aeruginosa* PA14 infection for 4 h, 8 h, and 12 h, worms were washed with M9 buffer for several times. After worms were homogenized in liquid nitrogen, the homogenate was lysed on ice for 30 minutes in lysis buffer (BioTeKe, Beijing, China). Following centrifugation at 10,000× g for 10 min, the supernatant was collected. Approximately 100 µg of total proteins per sample were submitted to Beijing Genomics Institute (BGI)-Shenzhen for proteomic analysis. Briefly, after trypsin digestion of the samples, the peptides were labeled with 4-Plex iTRAQ reagents (Applied Biosystems). Then the mixtures of iTRAQ-labeled peptides were fractionated into 10 portions using SCX chromatography (Shimadzu, Japan), and were subjected to nanoelectrospray ionization followed by tandem mass spectrometry (MS/MS) using the TripleTOF 5600 System (AB SCIEX, USA). Candidate proteins were quantified using ProteinPilot Software 4.0.8085 (Applied Biosystems-MDS SCIEX Ins). We set a 1.5-fold change as the threshold and a two-tailed *P*-value <0.05 to identify significant changes.

### Lifespan assays

Lifespan assays were conducted on NGM agar plates of *E. coli* OP50 at 20°C, starting with day 1 adults [43]. Animals were transferred to new plates during each day of their reproductive period and after that were transferred every third day. Survival of animals was monitored every day. Worms that did not move when gently prodded and displayed no pharyngeal pumping were marked as dead. Approximately 100 animals were tested on each plate with three replicates, and three independent assays were performed for each result.

### Statistics

These results are mean ± SD of three independent experiments performed in triplicate. Differences in survival rates were analyzed using the log-rank test. The statistical significance of differences in gene expression and fluorescence intensity was assessed by performing a one-way ANOVA followed by a Student-Newman-Keuls test. Data were analyzed using SPSS11.0 software (SPSS Inc.).

## Supporting Information

S1 Fig **Knockdown of** ***W03G11.4*** **by RNAi does not affect survival of worms after** ***Pseudomonas aeruginosa*** **PA14 infection.** (A) The strategy for knockdown of *W03G11.4* by RNAi. (B) qRT-PCR analysis of *W03G11.4* expression in wild type (WT) worms subjected to *W03G11.4* RNAi. *P<0.05 versus empty vector (EV). (C) Knockdown of *W03G11.4* by RNAi did not affect survival of WT worms after PA14 infection. (D) Knockdown of *F13H10.5* by RNAi enhanced resistance to PA14 infection. *P*<0.01 versus WT+EV.
(TIF)

S2 Fig ***mir-233(n4761)*** **mutants are hypersensitive to the killing by a variety of pathogenic bacteria.** (A) *Staphylococcus aureus*. *P*<0.01 versus wild type worms (WT). (B) *Enterococcus faecalis*. (C) *Salmonella typhimurium*. *P*<0.001 versus WT.
(TIF)

S3 Fig **A mutation in** ***mir-233(n4761)*** **does not affect lifespan of worms.** The *mir-233(n4761)* and wild type worms were grown on plates containing *E. coli* OP50. Lifespan was monitored every day.
(TIF)

S4 Fig **The putative target genes of** ***mir-233*** **.** MICRORNA, TargetScan, and DIANA-microT predict the 3′UTR of 1101, 277, and 284 genes, which contain predicted *mir-233* targeting sites, respectively. There were 38 overlap genes predicted by these miRNA target prediction algorithms.
(TIF)

S5 Fig **Knockdown of** ***sca-1*** **in a 1/4 dilution results in a decrease in the expression of** ***sca-1*** **.** qRT-PCR analysis of *sca-1* expression in wild type worms (WT) subjected to *sca-1* RNAi in a 1/4 dilution. *P<0.05 versus empty vector (EV).
(TIF)

S6 Fig ***P. aeruginosa*** **accumulation in the intestine of worms.** (A) Numbers of colony-forming units of *P. aeruginosa* PA14 were measured in the *mir-233(n4761)* mutant, or the *pmk-1(km25)* mutant, or worms subjected to *sca-1* RNAi. *P<0.05 versus WT. (B) Fluorescence of worms exposed to *P. aeruginosa* PA14 expressing GFP for 24 h. The image is representative of three independent experiments.
(TIF)

S7 Fig **ATF-7 is not involved in the induction of** ***mir-233*** **mediated by PMK-1.** (A) The expression of *mir-233* in the *atf-7(gk715)* mutant was comparable to that in wild type (WT) worms after *P. aeruginosa* PA14 infection. (B) *atf-7* RNAi did not influence the expression of *mir-233p::gfp* in WT and the *pmk-1(km25)* worms after PA14 infection.
(TIF)

S8 Fig **The** ***mir-233(n4761)*** **mutant on** ***P. aeruginosa*** **PA14 exhibits severely attenuated larval development.** Development of worms to the L4 larval stage or older after 3 days on *P. aeruginosa* PA14. **P<0.01 versus WT.
(TIF)

S9 Fig **A mutation in** ***xbp-1(zc12)*** **does not affect lifespan of worms.** The *xbp-1(zc12)* mutant and wild type worms were grown on plates containing *E. coli* OP50. Lifespan was monitored every day.
(TIF)

S10 Fig **PEK-1 is not involved in innate immune responses to** ***P. aeruginosa*** **infection.** The survival of the *pek-1(ok275)* mutant was comparable to that of wild type (WT) animals after *P. aeruginosa* PA14 infection at normal temperature (25°C).
(TIF)

S11 Fig **XBP-1 is a downstream effector in PMK-1-mediated innate immunity.** The survival of the *xbp-1;pmk-1* double mutants is comparable to that of the *pmk-1(km25)* mutant. *P<0.01 versus wild type worms (WT).
(TIF)

S12 Fig ***mir-233*** **is mainly expressed in the intestine.** Transgenic animals expressing *mir-233p::gfp* were observed under a fluorescence microscope.
(TIF)

S13 Fig **Epidermal- or muscular-specific** ***sca-1*** **RNAi does not affect on sensitivity to** ***P. aeruginosa*** **PA14 infection.** (A–B) NR222 strains (A) and NR350 strains (B) subjected to *sca-1* RNAi in a 1/4 dilution were exposed to *P. aeruginosa* PA14.
(TIF)

S14 Fig **Epidermal- or muscular-specific** ***xbp-1*** **RNAi does not affect on sensitivity to** ***P. aeruginosa*** **PA14 infection.** (A–B) NR222 strains (A) and NR350 strains (B) subjected to *xbp-1* RNAi were exposed to *P. aeruginosa* PA14.
(TIF)

S1 Table **The expression of miRNAs is up-regulated by** ***Pseudomonas aeruginosa*** **infection.**
(DOC)

S2 Table **miRNAs are involved in innate immune responses to** ***P. aeruginosa*** **PA14 infection.**
(DOC)

S3 Table **The expression of proteins is down-regulated or up-regulated at 4 h post-infection.**
(DOC)

S4 Table **The expression of proteins is down-regulated or up-regulated at 8 h post-infection.**
(DOC)

S5 Table **The expression of proteins is down-regulated or up-regulated at 12 h post-infection.**
(DOC)

S6 Table **The miRNA mutants in this study.**
(DOC)
