# Supplementary figures and images for: mir-233 Modulates the Unfolded Protein Response in C. elegans during Pseudomonas aeruginosa Infection
Source: PLoS Pathog. 2015 Jan 8;11(1):e1004606. doi: 10.1371/journal.ppat.1004606 (PMC4287614; doi:10.1371/journal.ppat.1004606)

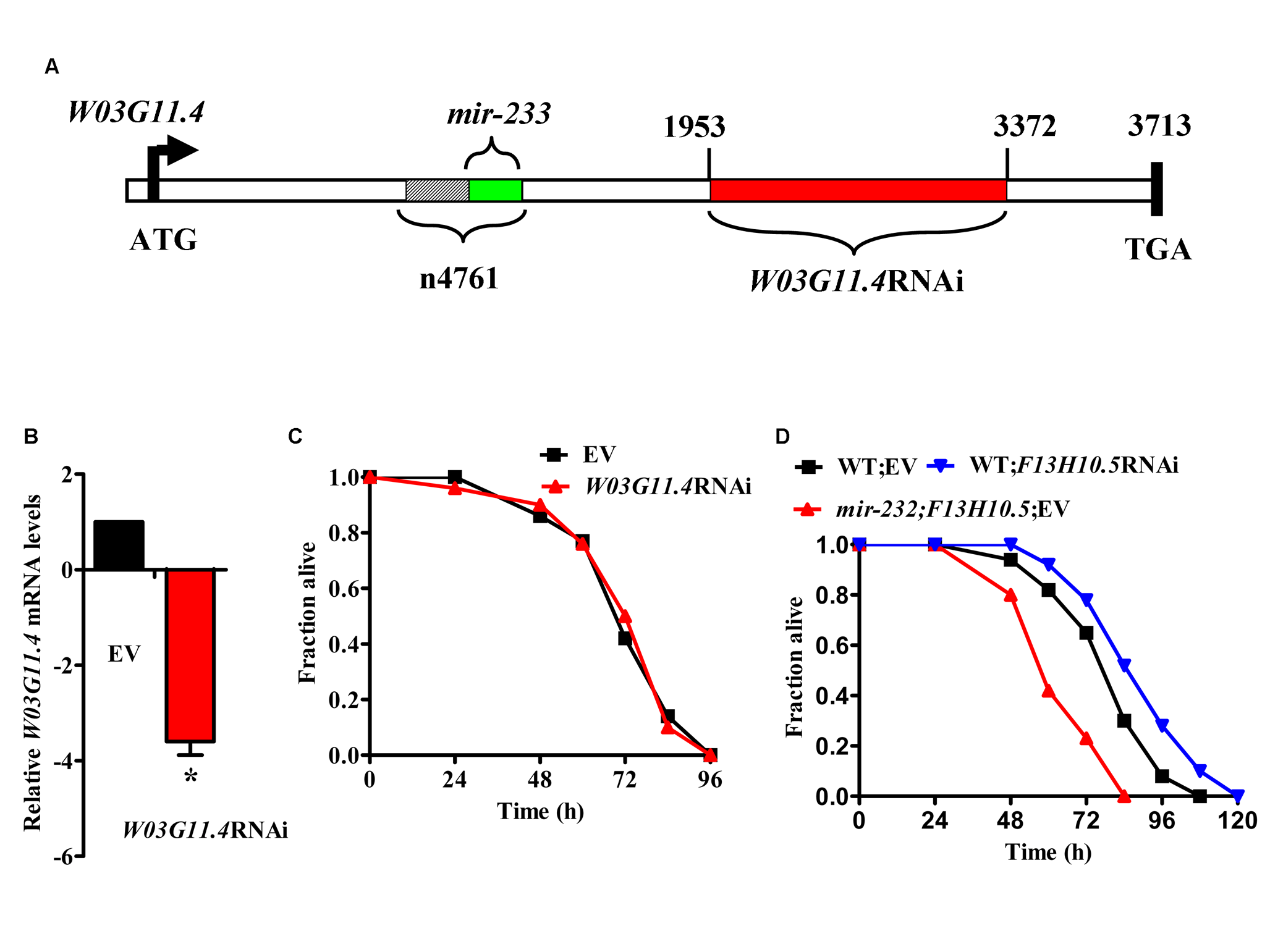

Supplement: S1 Fig — Knockdown of W03G11.4 by RNAi does not affect survival of worms after Pseudomonas aeruginosa PA14 infection. (A) The strategy for knockdown of W03G11.4 by RNAi. (B) qRT-PCR analysis of W03G11.4 expression in wild type (WT) worms subjected to W03G11.4 RNAi. *P<0.05 versus empty vector (EV). (C) Knockdown of W03G11.4 by RNAi did not affect survival of WT worms after PA14 infection. (D) Knockdown of F13H10.5 by RNAi enhanced resistance to PA14 infection. P<0.01 versus WT+EV. (TIF) [file ppat.1004606.s001.tif]

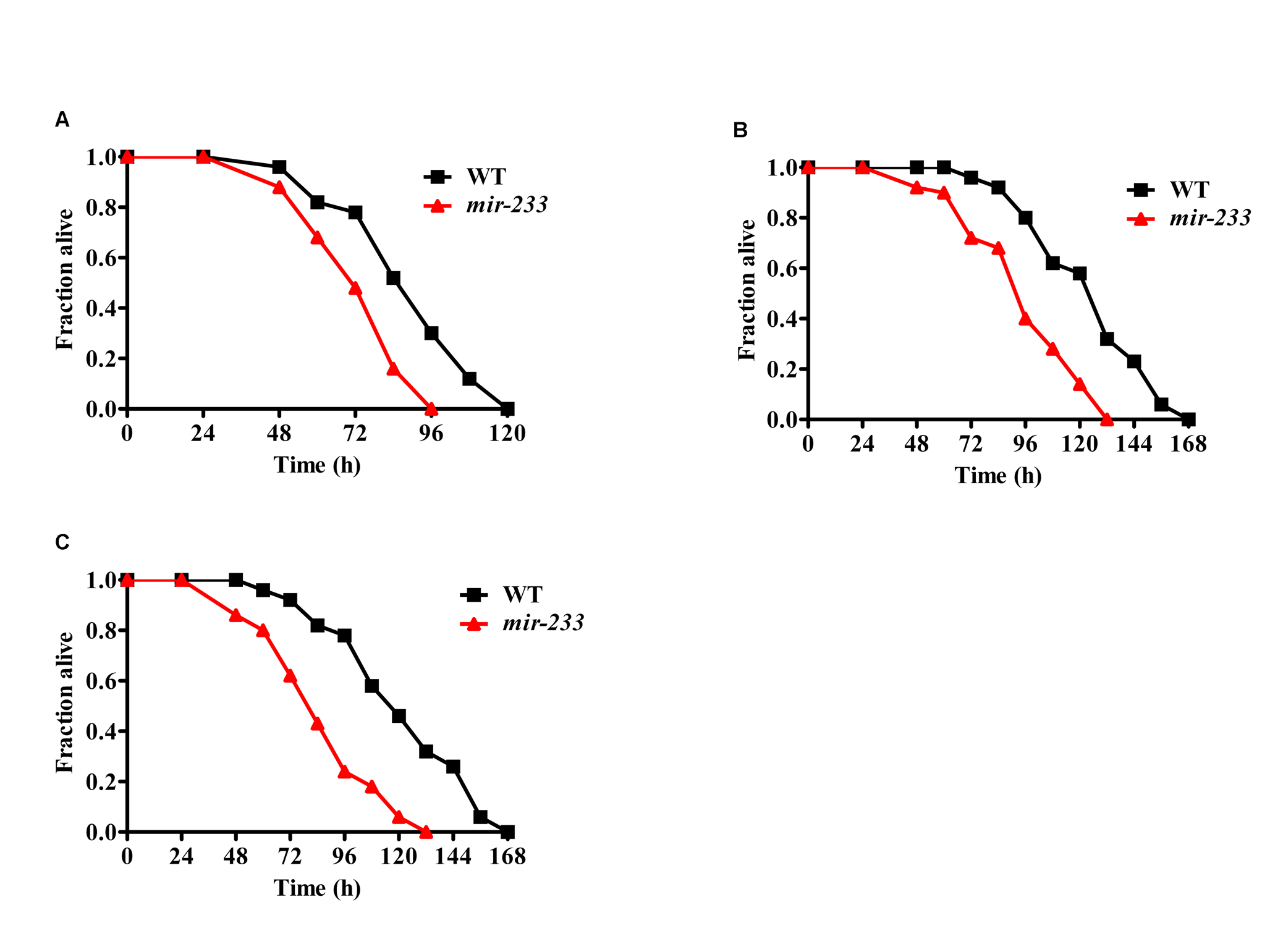

Supplement: S2 Fig — mir-233(n4761) mutants are hypersensitive to the killing by a variety of pathogenic bacteria. (A) Staphylococcus aureus. P<0.01 versus wild type worms (WT). (B) Enterococcus faecalis. (C) Salmonella typhimurium. P<0.001 versus WT. (TIF) [file ppat.1004606.s002.tif]

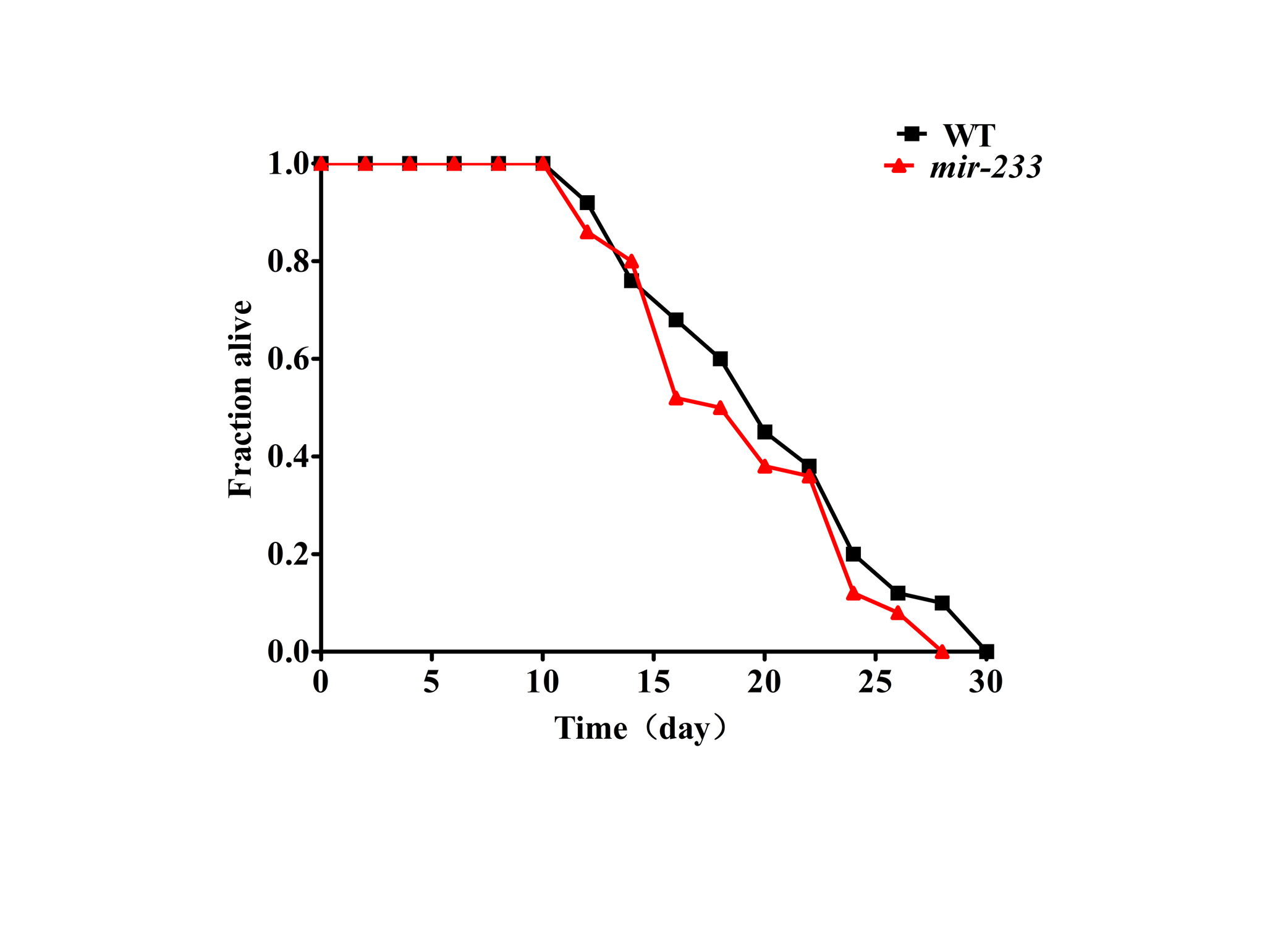

Supplement: S3 Fig — A mutation in mir-233(n4761) does not affect lifespan of worms. The mir-233(n4761) and wild type worms were grown on plates containing E. coli OP50. Lifespan was monitored every day. (TIF) [file ppat.1004606.s003.tif]

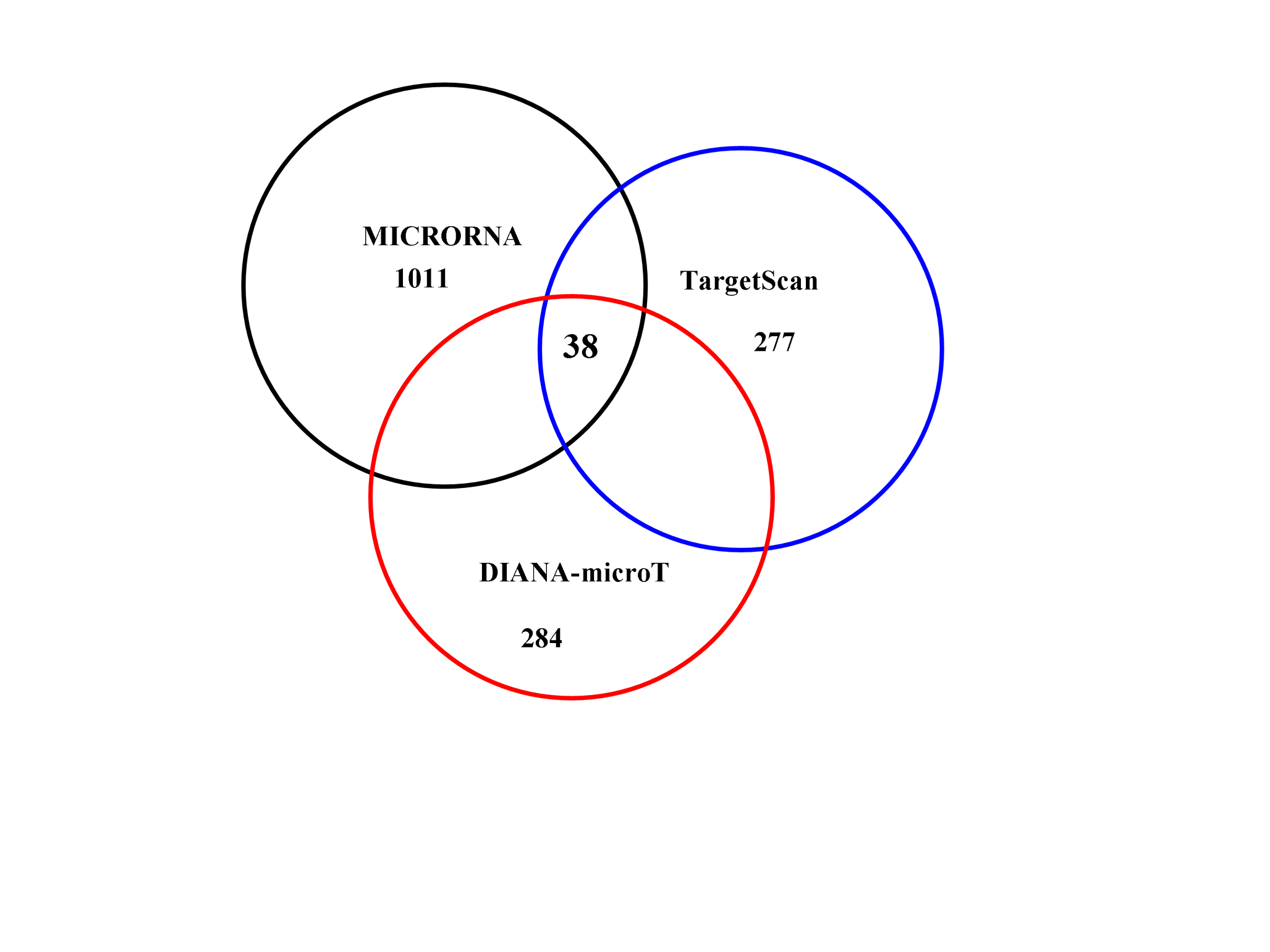

Supplement: S4 Fig — The putative target genes of mir-233 . MICRORNA, TargetScan, and DIANA-microT predict the 3′UTR of 1101, 277, and 284 genes, which contain predicted mir-233 targeting sites, respectively. There were 38 overlap genes predicted by these miRNA target prediction algorithms. (TIF) [file ppat.1004606.s004.tif]

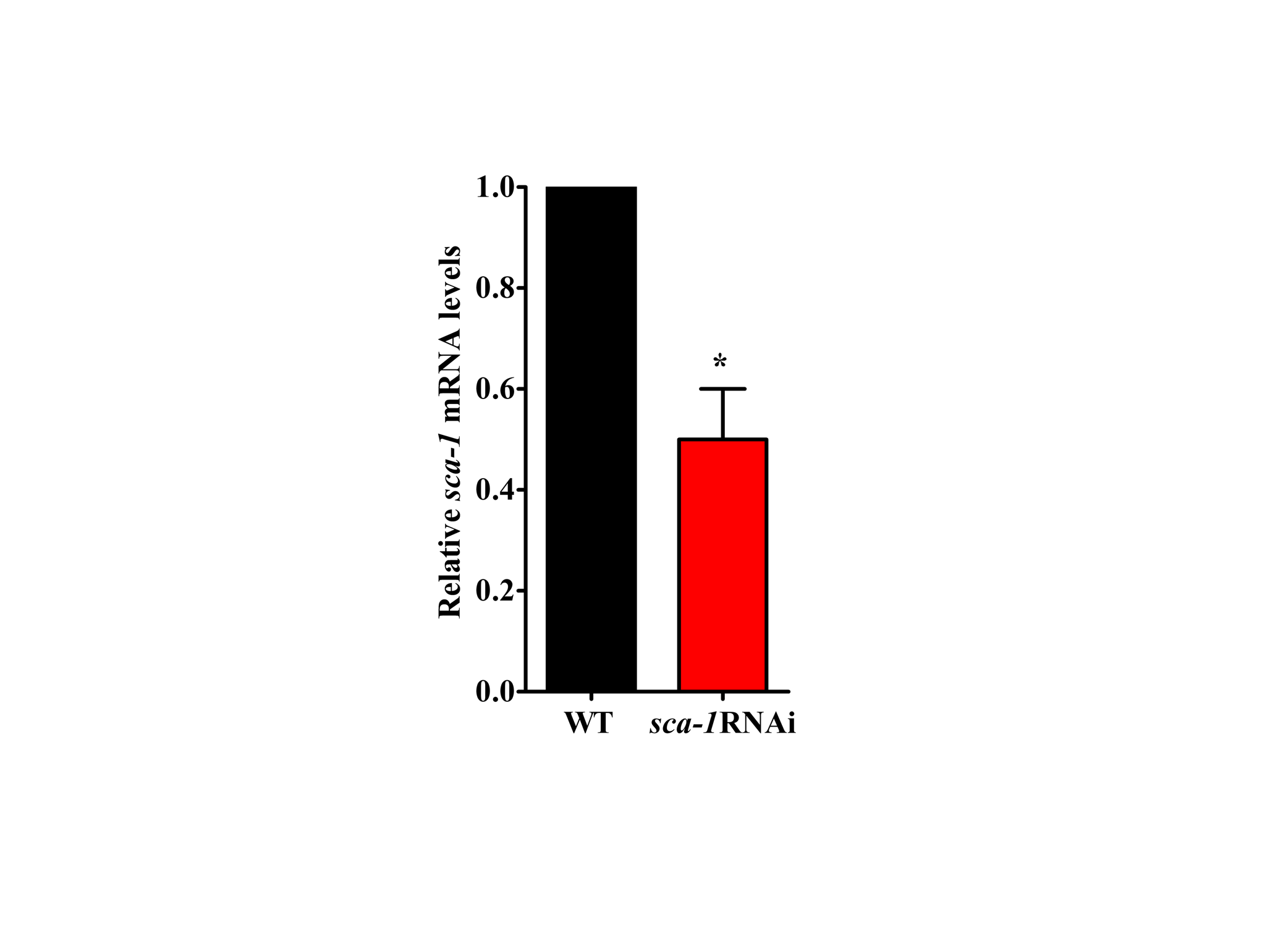

Supplement: S5 Fig — Knockdown of sca-1 in a 1/4 dilution results in a decrease in the expression of sca-1 . qRT-PCR analysis of sca-1 expression in wild type worms (WT) subjected to sca-1 RNAi in a 1/4 dilution. *P<0.05 versus empty vector (EV). (TIF) [file ppat.1004606.s005.tif]

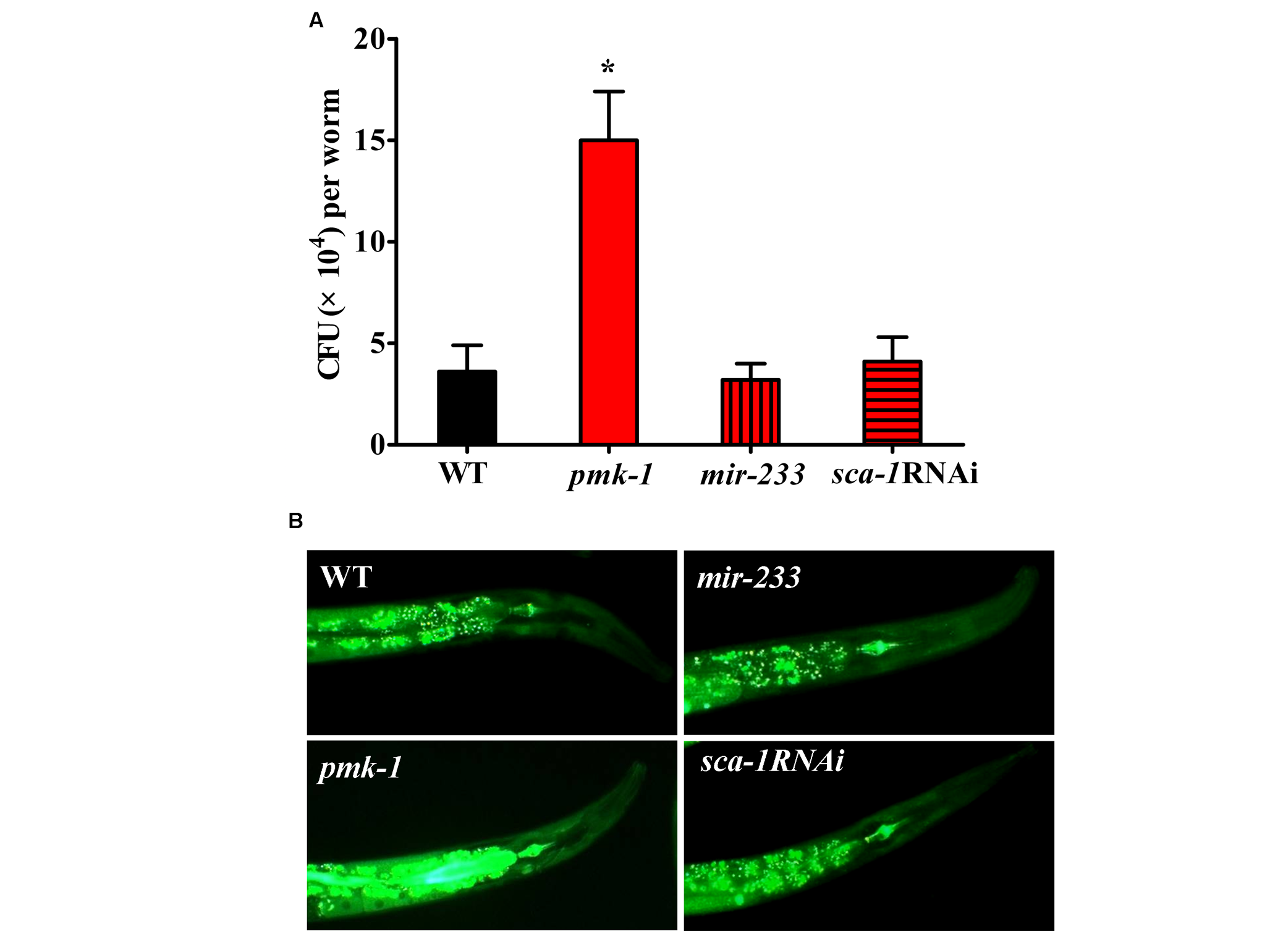

Supplement: S6 Fig — P. aeruginosa accumulation in the intestine of worms. (A) Numbers of colony-forming units of P. aeruginosa PA14 were measured in the mir-233(n4761) mutant, or the pmk-1(km25) mutant, or worms subjected to sca-1 RNAi. *P<0.05 versus WT. (B) Fluorescence of worms exposed to P. aeruginosa PA14 expressing GFP for 24 h. The image is representative of three independent experiments. (TIF) [file ppat.1004606.s006.tif]

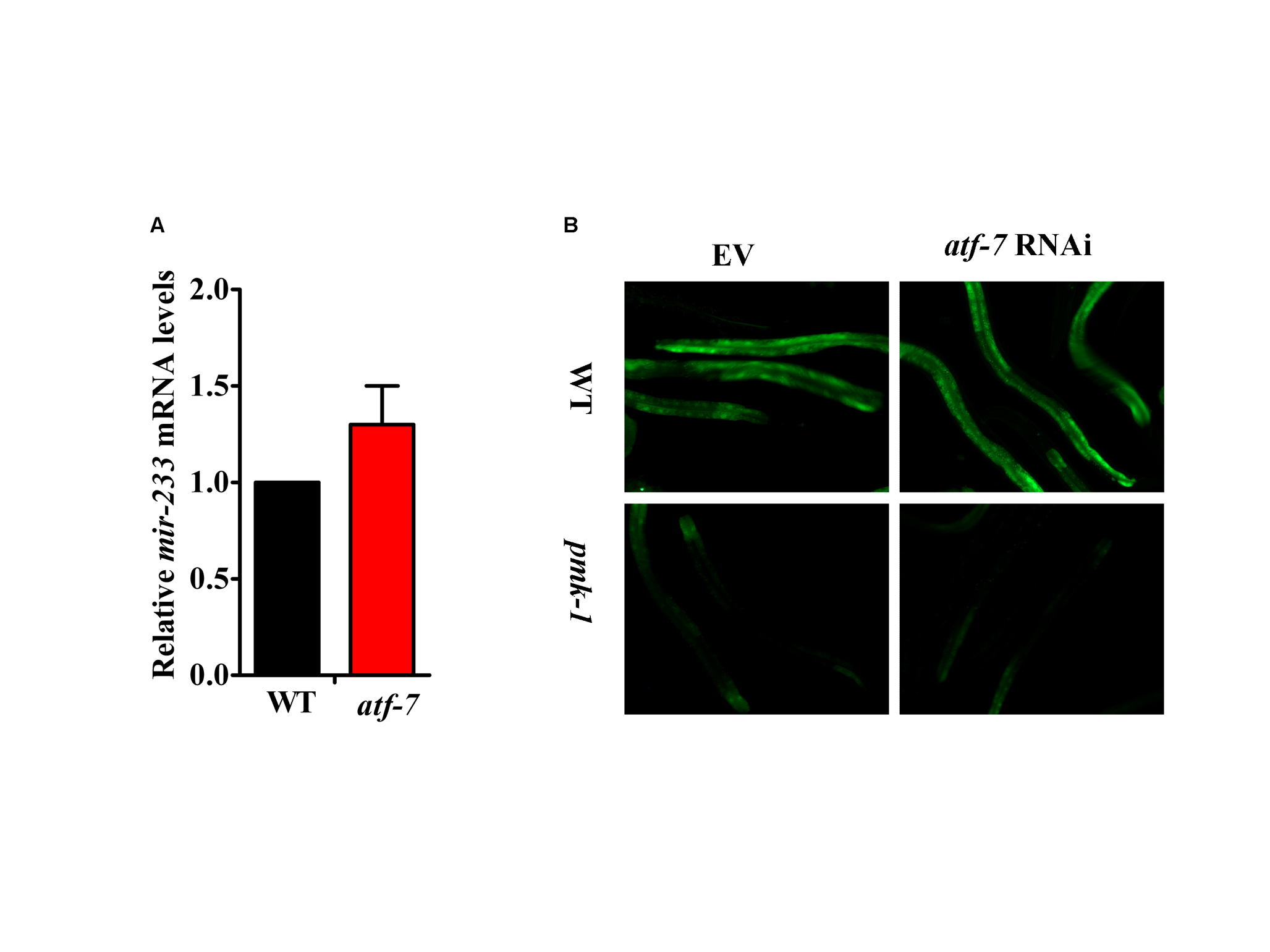

Supplement: S7 Fig — ATF-7 is not involved in the induction of mir-233 mediated by PMK-1. (A) The expression of mir-233 in the atf-7(gk715) mutant was comparable to that in wild type (WT) worms after P. aeruginosa PA14 infection. (B) atf-7 RNAi did not influence the expression of mir-233p::gfp in WT and the pmk-1(km25) worms after PA14 infection. (TIF) [file ppat.1004606.s007.tif]

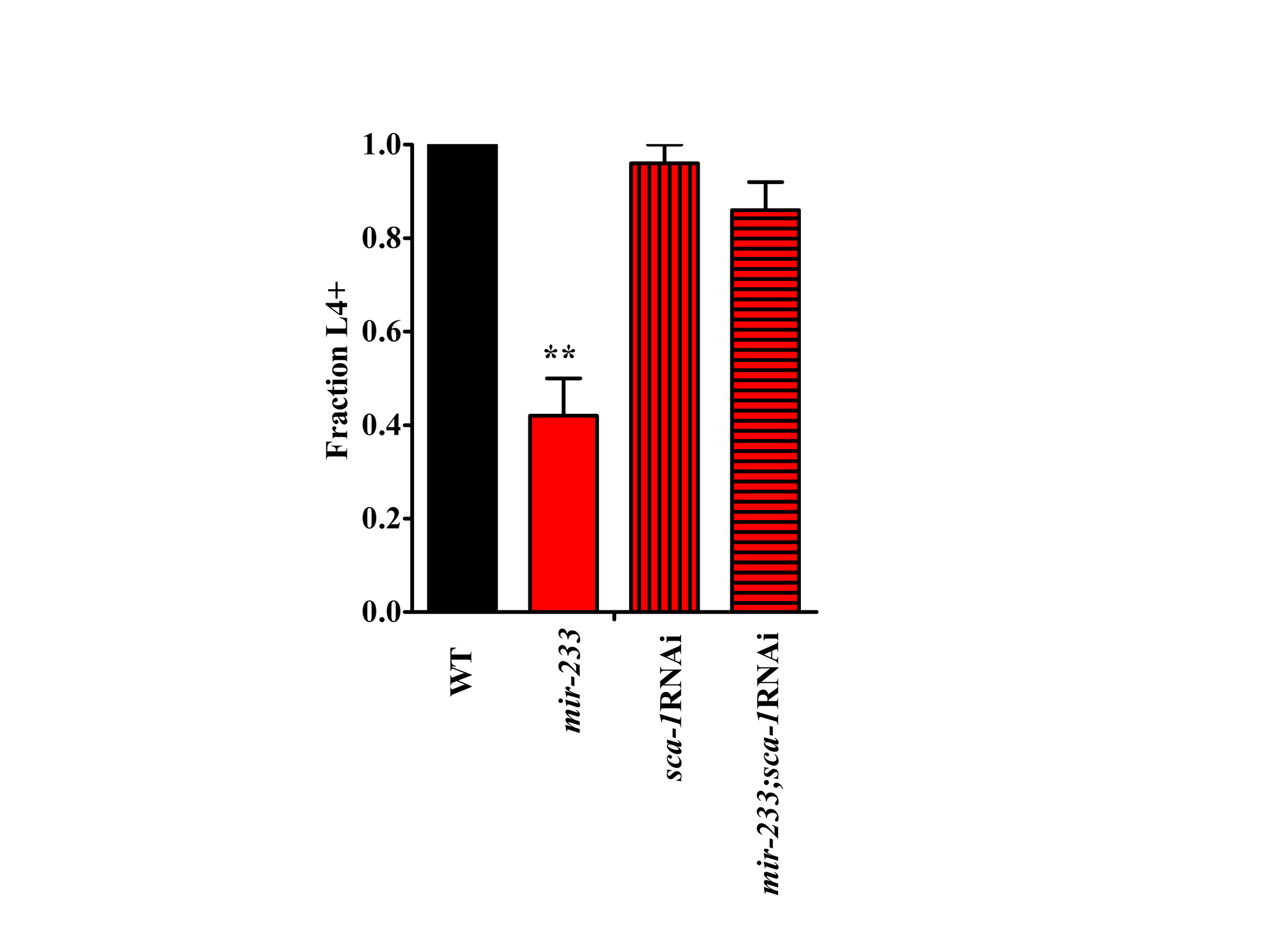

Supplement: S8 Fig — The mir-233(n4761) mutant on P. aeruginosa PA14 exhibits severely attenuated larval development. Development of worms to the L4 larval stage or older after 3 days on P. aeruginosa PA14. **P<0.01 versus WT. (TIF) [file ppat.1004606.s008.tif]

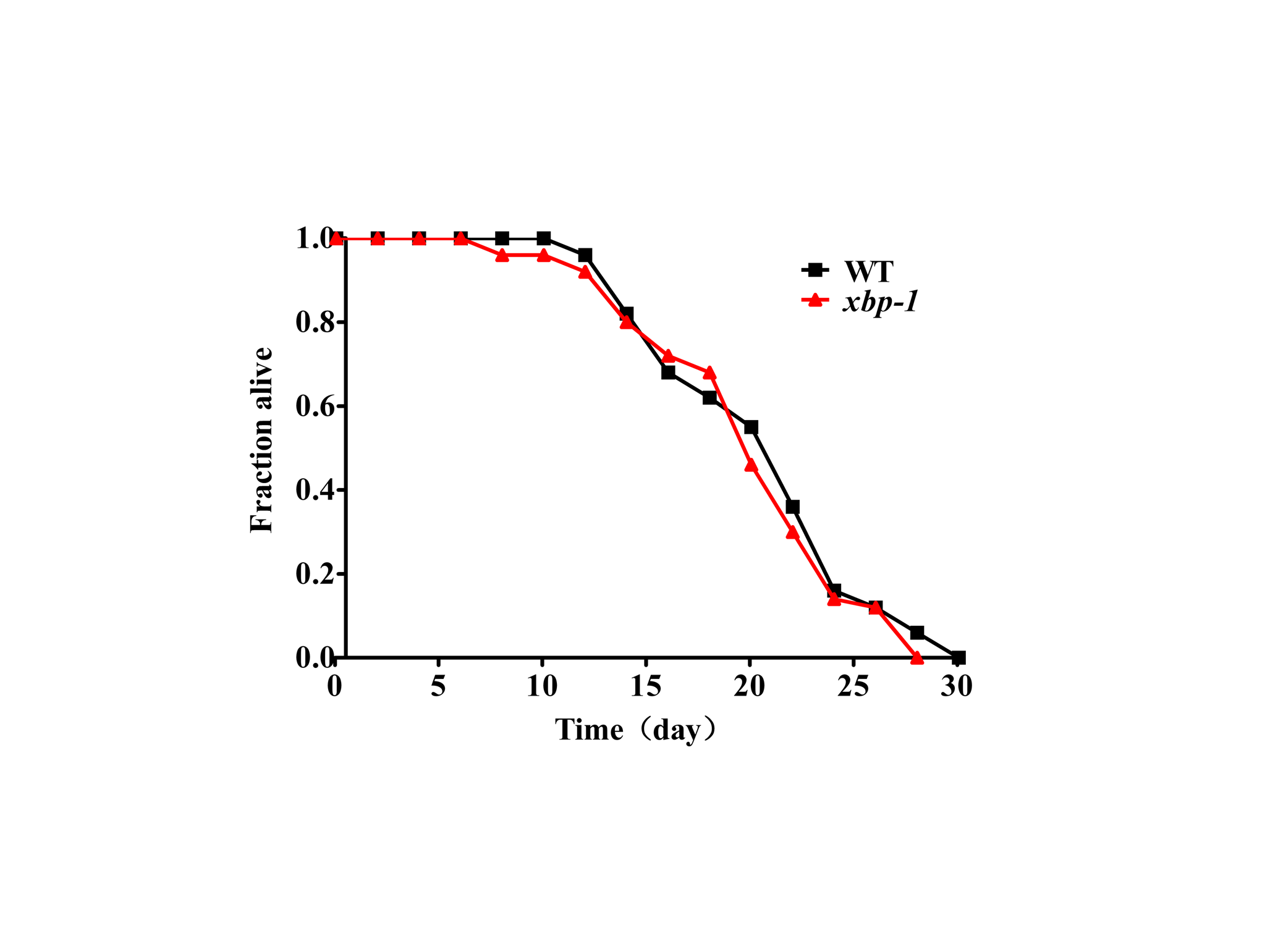

Supplement: S9 Fig — A mutation in xbp-1(zc12) does not affect lifespan of worms. The xbp-1(zc12) mutant and wild type worms were grown on plates containing E. coli OP50. Lifespan was monitored every day. (TIF) [file ppat.1004606.s009.tif]

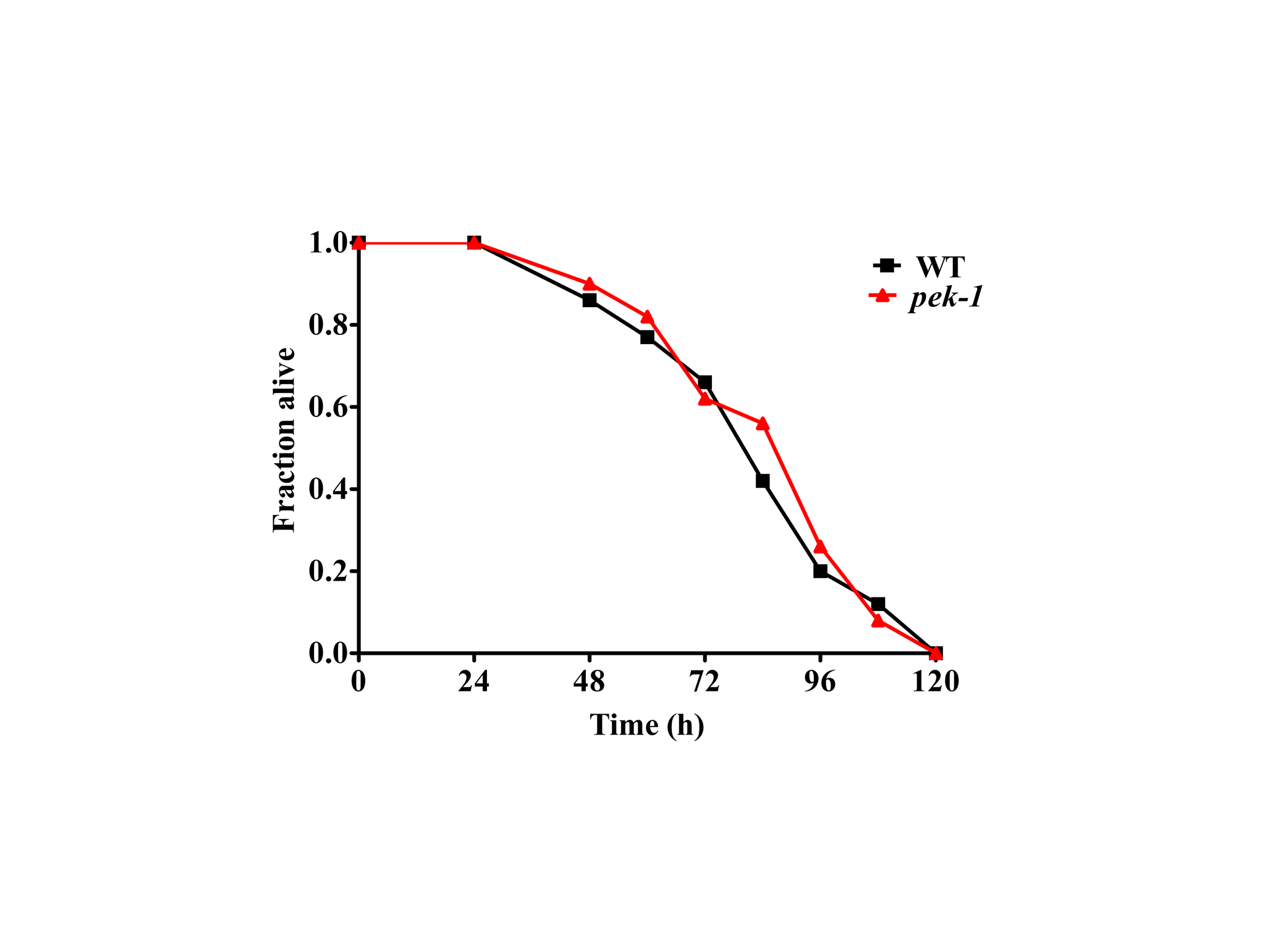

Supplement: S10 Fig — PEK-1 is not involved in innate immune responses to P. aeruginosa infection. The survival of the pek-1(ok275) mutant was comparable to that of wild type (WT) animals after P. aeruginosa PA14 infection at normal temperature (25°C). (TIF) [file ppat.1004606.s010.tif]

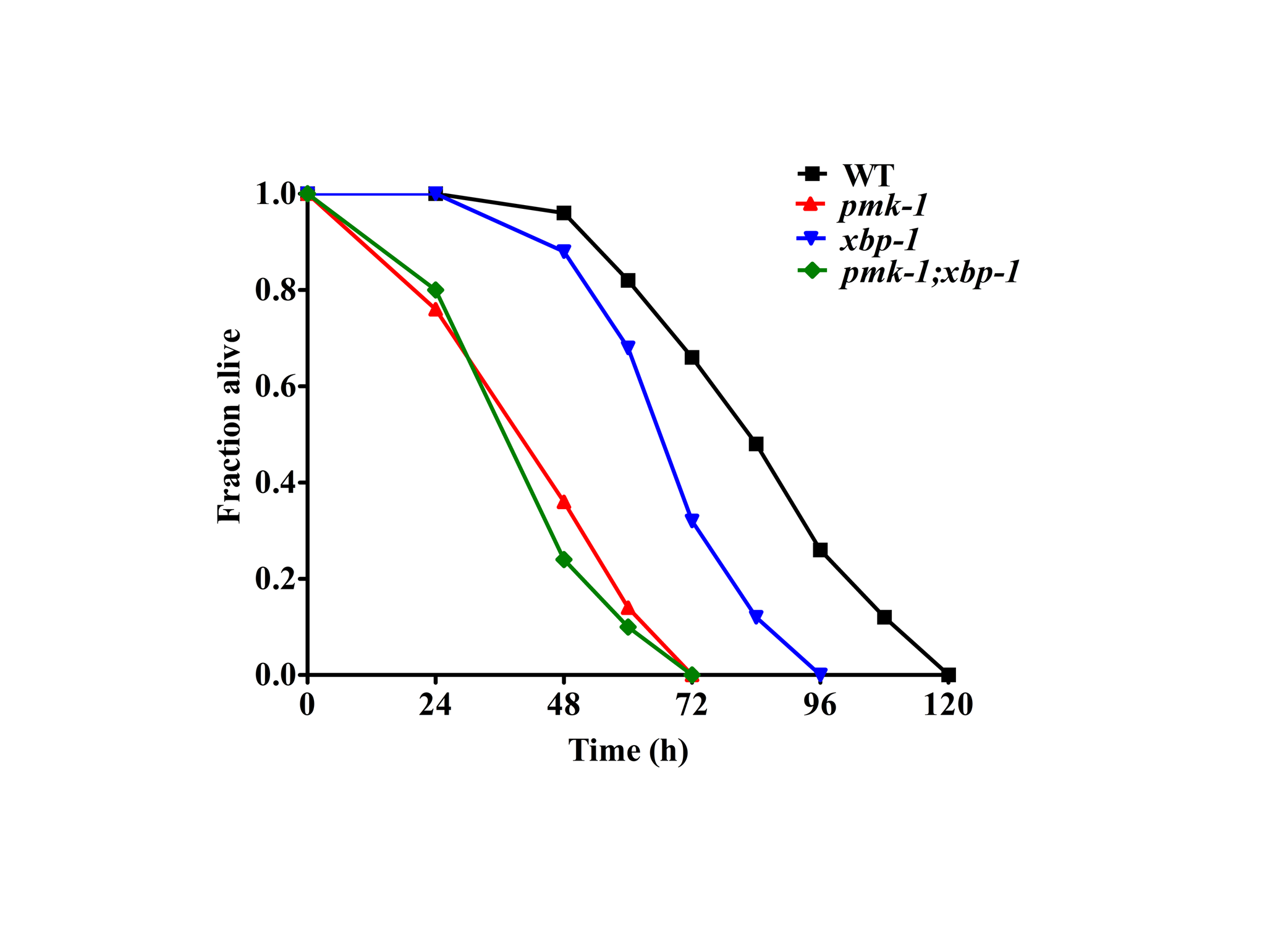

Supplement: S11 Fig — XBP-1 is a downstream effector in PMK-1-mediated innate immunity. The survival of the xbp-1;pmk-1 double mutants is comparable to that of the pmk-1(km25) mutant. *P<0.01 versus wild type worms (WT). (TIF) [file ppat.1004606.s011.tif]

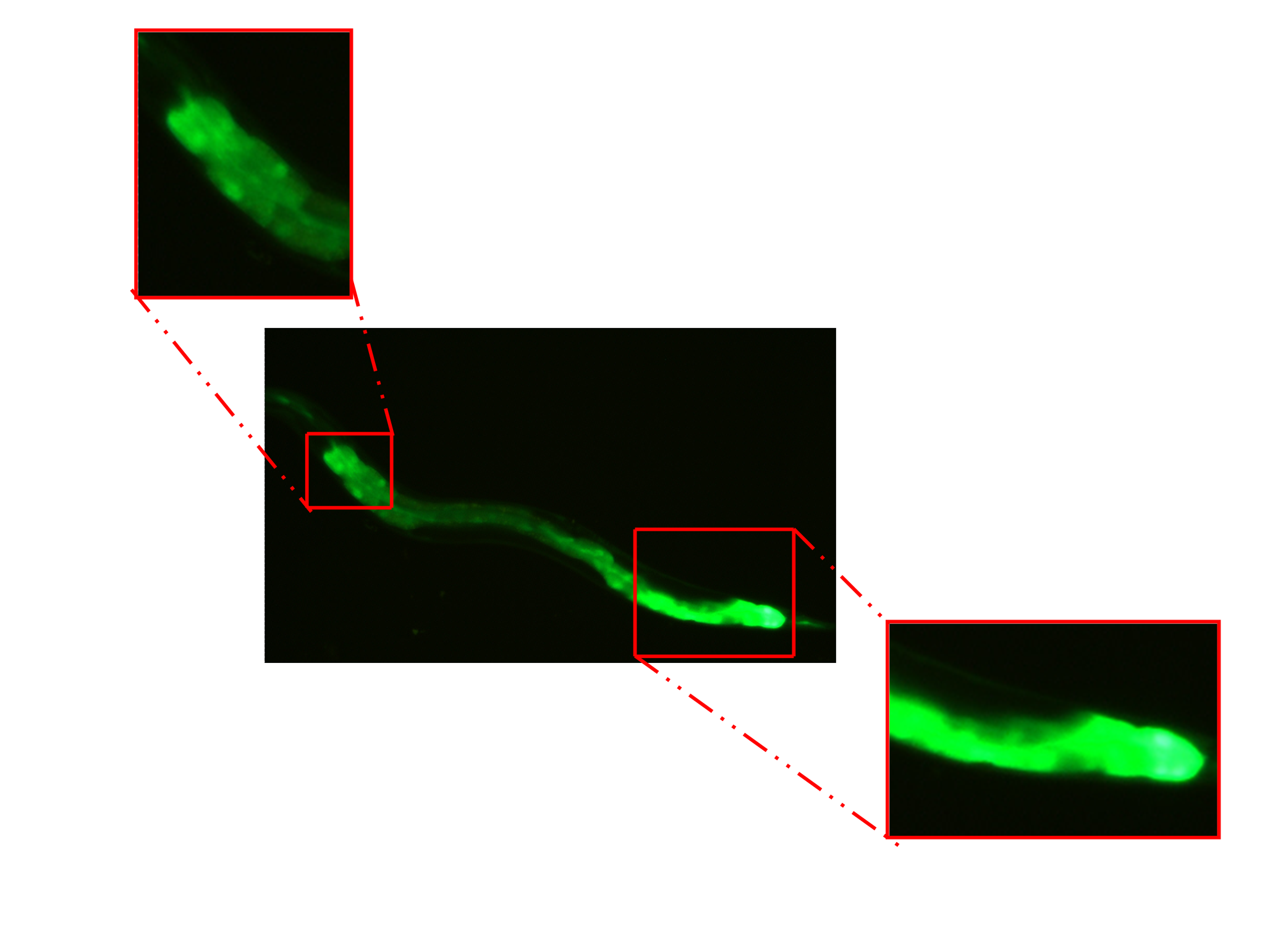

Supplement: S12 Fig — mir-233 is mainly expressed in the intestine. Transgenic animals expressing mir-233p::gfp were observed under a fluorescence microscope. (TIF) [file ppat.1004606.s012.tif]

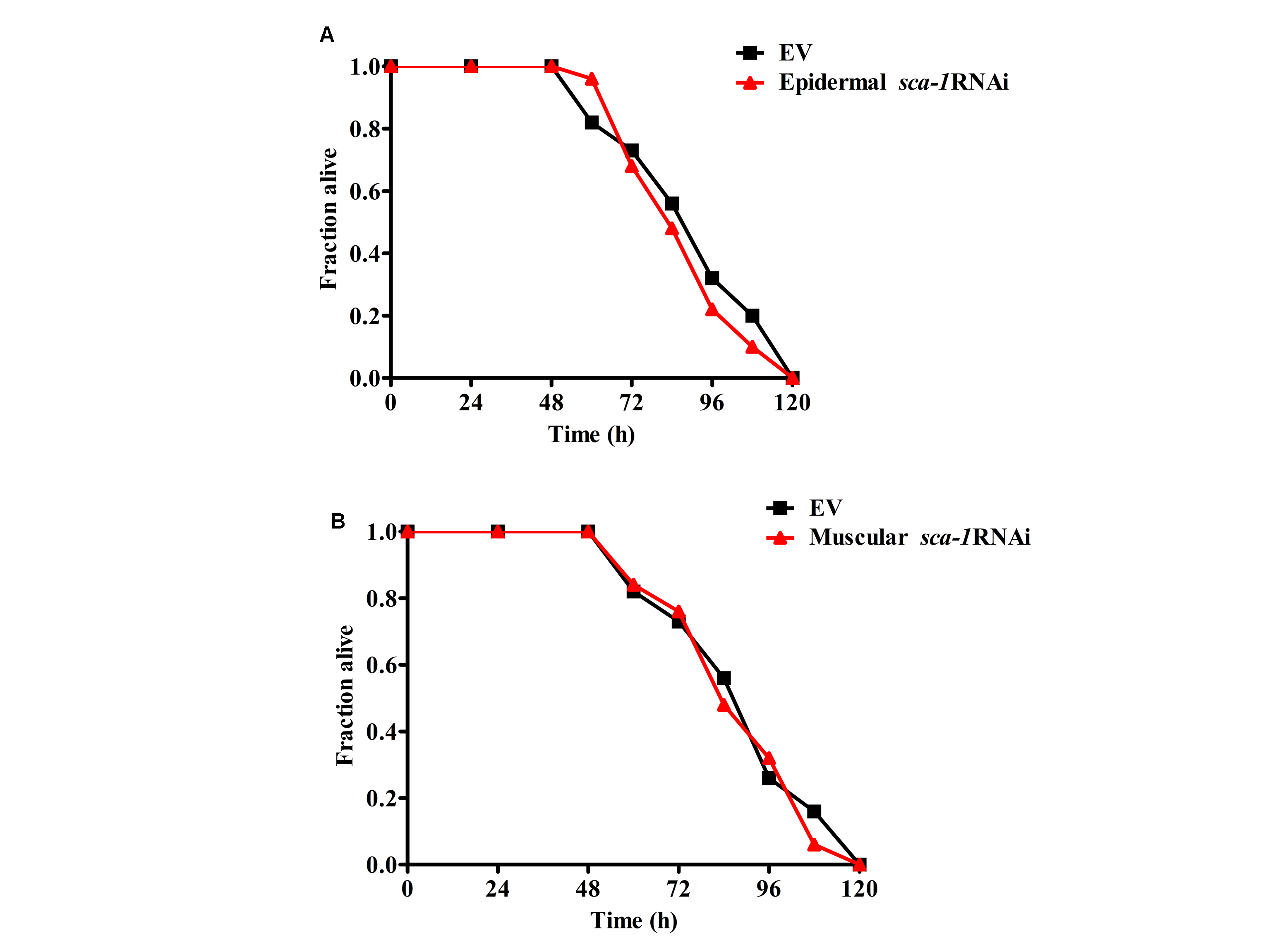

Supplement: S13 Fig — Epidermal- or muscular-specific sca-1 RNAi does not affect on sensitivity to P. aeruginosa PA14 infection. (A–B) NR222 strains (A) and NR350 strains (B) subjected to sca-1 RNAi in a 1/4 dilution were exposed to P. aeruginosa PA14. (TIF) [file ppat.1004606.s013.tif]

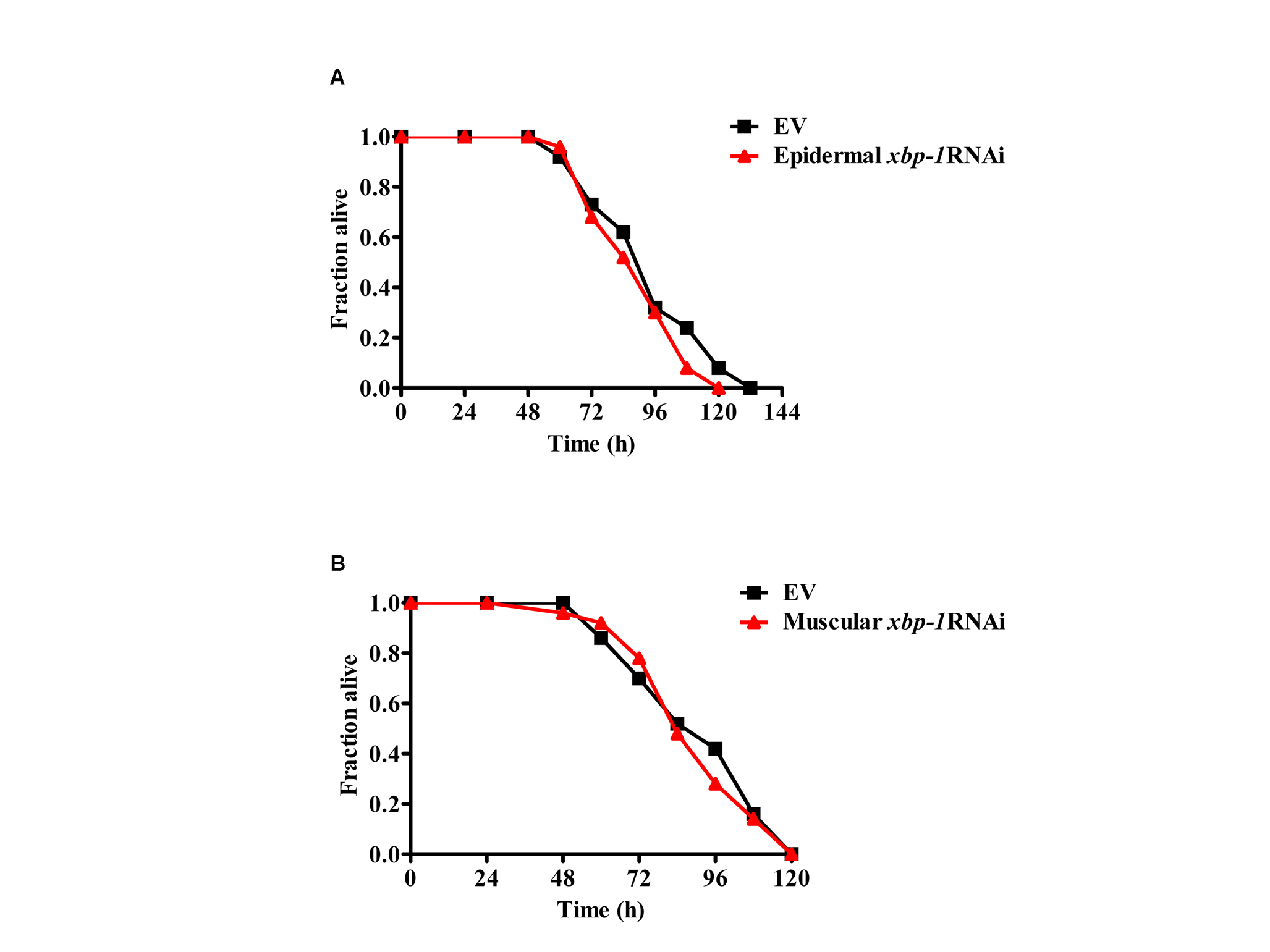

Supplement: S14 Fig — Epidermal- or muscular-specific xbp-1 RNAi does not affect on sensitivity to P. aeruginosa PA14 infection. (A–B) NR222 strains (A) and NR350 strains (B) subjected to xbp-1 RNAi were exposed to P. aeruginosa PA14. (TIF) [file ppat.1004606.s014.tif]
