# Supplementary material for: mir-233 Modulates the Unfolded Protein Response in C. elegans during Pseudomonas aeruginosa Infection
Source: PLoS Pathog. 2015 Jan 8;11(1):e1004606. doi: 10.1371/journal.ppat.1004606 (PMC4287614; doi:10.1371/journal.ppat.1004606)
Supplement: S1 Table — The expression of miRNAs is up-regulated by Pseudomonas aeruginosa infection. (DOC) [file ppat.1004606.s015.doc]

**Table S1 The expression of miRNAs was up-regulated by *Pseudomonas aeruginosa* infection**

| **miRNA name** | **Fold change (log_2_)** |  |
| --- | --- | --- |
|  | **4 h 8 h 12h** |  |
| mir-124-5p 2.1987 1.6951 2.2904 | | |
| mir-124-3p 5.116 | | |
| mir-1-5p 1.3974 1.4321 2.1174 | | |
| mir-2217-3p 4.1461 | | |
| mir-232-3p 1.711 1.5058 1.6483 | | |
| mir-233-3p 1.0918 | | |
| mir-235-3p 2.1847 2.1428 2.2304 | | |
| mir-235-5p 1.5604 1.0488 | | |
| mir-238-3p 1.192 1.7188 1.9829 | | |
| mir-239a-5p 1.9592 2.5158 2.6888 | | |
| mir-239b-5p 2.0079 3.6394 3.0355 | | |
| mir-2-3p 1.8691 1.6267 1.8145 | | |
| mir-241-3p 2.0628 2.432 2.5191 | | |
| mir-243-5p 1.1198 1.9284 1.8563 | | |
| mir-245-3p 1.5755 1.1858 1.4845 | | |
| mir-259-5p 2.9494 2.5135 2.5054 | | |
| mir-34-3p 1.6245 3.6676 1.9807 | | |
| mir-47-3p 1.3965 1.2305 2.0988 | | |
| mir-48-3p 1.7647 1.8823 | | |
| mir-49-3p 1.4213 1.2903 1.2747 | | |
| mir-51-3p 1.0685 1.4279 | | |
| mir-52-5p 1.5914 1.1685 | | |
| mir-53-5p 1.7717 1.2715 1.6827 | | |
| mir-54-3p 1.6998 1.4244 2.4319 | | |
| mir-56-5p 1.8239 1.695 2.0127 | | |
| mir-60-3p 1.5906 1.7355 1.7963 | | |
| mir-65-3p 1.3677 3.2734 1.3127 | | |
| mir-66-5p 1.3235 1.679 1.4022 | | |
| mir-67-3p 1.0706 1.1708 | | |
| mir-73-5p 1.5604 2.9175 2.6827 | | |
| mir-74-3p 2.157 1.6565 2.1441 | | |
| mir-75-3p 1.7447 1.3434 1.529 | | |
| mir-77-3p 1.5396 2.1709 2.261 | | |
| mir-785 1.1471 1.0862 | | |
| mir-788-5p 1.9575 1.9086 2.3826 | | |
| mir-792-3p 2.3904 | | |
| mir-79-3p 1.3188 1.2275 1.4556 | | |
| mir-798 1.1819 2.2171 2.1057 | | |
| mir-83-3p 1.2821 1.3459 1.4829 | | |
| mir-86-5p 2.8083 1.7473 1.9817 | | |
| mir-87-3p 2.4483 2.4199 2.6801 | | |
| let-7-3p 4.5698 5.7582 | | |
| let-7-5p 1.8053 3.3481 | | |
| lys-6 1.081 | | |
| mir-1822-3p 1.4728 | | |
| mir-1829c 1.1102 1.1211 | | |
| mir-2214-3p 1.0431 1.8338 | | |
| mir-239a-3p 2.3511 2.0305 | | |
| mir-239b-3p 6.7138 | | |
| mir-240-3p 4.5032 6.7464 | | |
| mir-250-5p 1.9402 1.4868 | | |
| mir-34-5p 1.1679 | | |
| mir-42-5p 1.3458 | | |
| mir-45-5p 1.6879 | | |
| mir-46-3p 1.0322 | | |
| mir-4813-5p 1.83 | | |
| mir-55-5p 1.6681 1.26 | | |
| mir-58-5p 1.4023 1.792 | | |
| mir-59-3p 1.4908 1.2972 | | |
| mir-243-3p 1.6854 1.3593 | | |
| mir-248 1.1672 | | |
| mir-60-5p 1.7673 | | |
| mir-61-5p 1.1261 | | |
| mir-62 1.3527 | | |
| mir-63-3p 1.4088 1.2403 | | |
| mir-63-5p 1.207 | | |
| mir-64-5p 1.0694 1.4242 | | |
| mir-71-3p 1.1647 | | |
| mir-71-5p 1.1938 | | |
| mir-72-3p 1.5462 1.5692 | | |
| mir-794-5p 1.3005 1.3973 | | |
| mir-797-5p 1.5956 2.2037 | | |
| mir-81-5p 1.0717 | | |
| mir-84-5p 1.0693 | | |
| mir-229-5p 1.3486 | | |
| mir-247-3p 1.1141 | | |
| mir-46-5p 1.0111 | | |
| mir-4816-3p 1.4603 | | |
| mir-4929 1.9629 | | |
| mir-52-3p 1.5973 | | |
| mir-53-3p 1.5973 | | |
| mir-66-3p 1.3161 | | |
| mir-75-5p 1.6407 | | |
| mir-786-3p 3.8291 | | |
| mir-789 1.1779 | | |
| mir-795-3p 1.4603 | | |
| mir-79-5p 1.2289 | | |
| mir-85-3p 4.3344 | | |
