# Supplementary material for: mir-233 Modulates the Unfolded Protein Response in C. elegans during Pseudomonas aeruginosa Infection
Source: PLoS Pathog. 2015 Jan 8;11(1):e1004606. doi: 10.1371/journal.ppat.1004606 (PMC4287614; doi:10.1371/journal.ppat.1004606)
Supplement: S2 Table — miRNAs are involved in innate immune responses to P. aeruginosa PA14 infection. (DOC) [file ppat.1004606.s016.doc]

**Table S2 miRNAs are involved in innate immune responses**

**to *P. aeruginosa* PA14 infection**

| miRNAs | TD mean (h)  (Mean±SEM, n=3) | *p-value* |
| --- | --- | --- |
| WT | 59.2±3.9 |  |
| mir-84(n4037) | 67.7±3.5 |  |
| mir-232(nDF56) | 31.3±3.8 | <0.01 |
| mir-67(n4899) | 61.7±4.7 |  |
| mir-86(n4607) | 58.8±3.3 |  |
| mir-85(n4117) | 57.3±3.7 |  |
| mir-63(n4568) | 62.7±4.1 |  |
| mir-243(n4759) | 58.2±5.0 |  |
| mir-233(n4761) | 32.6±3.3 | <0.01 |
| mir-245(n4798) | 61.5±2.6 |  |
| mir-62(n4539) | 60.2±3.4 |  |
| mir-259(n4106) | 59.1±2.1 |  |
| mir-34(n4276) | 58.3±4.1 |  |
| mir-83(n4638) | 59.0±2.8 |  |
| mir-87(n4104) | 55.7±4.9 |  |
| mir-45(n4280) | 60.7±4.5 |  |
| mir-53(n4113) | 60.8±3.8 |  |
| mir-51(n4473) | 57.3±4.8 |  |
| mir-71(n4115) | 58.9±4.3 |  |
| mir-72(n4130) | 59.3±2.0 |  |
| mir-1(n4102) | 60.4±2.3 |  |
| Let-7(n2853) | 56.2±3.3 |  |
| mir-58(4640) | 69.2±4.3 |  |
| Lys-6(ot71) | 59.7±3.7 |  |
| mir-79(n4126) | 63.6±4.6 |  |
| mir-52(n4100) | 60.1±3,2 |  |
| mir-124(n4255) | 57.9±2.9 |  |
| mir-1(n4101) | 61.2±4.6 |  |
| mir-235(n4504) | 58.7±3.7 |  |
| mir-75(4471) | 68.7±4.5 |  |
| mir-77(n4286) | 59.6±3.2 |  |
| mir-2(n4108) | 59.1±4.9 |  |
| mir-59(n4604) | 60.1±3.1 |  |
| mir-46(n4475) | 62.1±2.3 |  |
| mir-47(gk167) | 61.7±3.8 |  |
| mir-60(n4947) | 62.2±4.3 |  |
| mir-238(n4112) | 63.1±3.0 |  |
| mir-239(nDF62) | 57.9±2.8 |  |
| mir-48,mir-241(nDF51) | 59.3±3.7 |  |
| mir-81-82(nDF54) | 65.1±4.3 |  |
| mir-54-56(nDF58) | 57.2±3.8 |  |
| mir-64,mir-229(nDF52) | 61.2±4.7 |  |
| mir-64-66,mir-229(nDF63) | 57.8±3.5 |  |
| mir-73-74(nDF47) | 62.7±3.8 |  |
| mir-247, mir-797(n4505) | 58.6±3.3 |  |
| mir-42-44(nDF49) | 61.7±4.3 |  |
| mir-61, mir-250(nDF59) | 56.2±3.8 |  |
| mir-240,mir-786(n4541) | 59.9±2.8 |  |
