# Supplementary material for: mir-233 Modulates the Unfolded Protein Response in C. elegans during Pseudomonas aeruginosa Infection
Source: PLoS Pathog. 2015 Jan 8;11(1):e1004606. doi: 10.1371/journal.ppat.1004606 (PMC4287614; doi:10.1371/journal.ppat.1004606)
Supplement: S3 Table — The expression of proteins is down-regulated or up-regulated at 4 h post-infection. (DOC) [file ppat.1004606.s017.doc]

**Table S3** **The expression of proteins was down-regulated**

**or up-regulated at 4 h post-infection**

| **Gene name** | **Fold change** |
| --- | --- |
| rpl-2 | 0.407 |
| F15E11.15 | 0.414 |
| rps-24 | 0.416 |
| pqn-59 | 0.458 |
| rpl-19 | 0.466 |
| Y37E3.8 | 0.482 |
| ndk-1 | 0.501 |
| cey-4 | 0.508 |
| rpl-34 | 0.57 |
| gpdh-2 | 0.578 |
| rpl-43 | 0.582 |
| cgh-1 | 0.617 |
| rpl-34 | 0.617 |
| W06H3.3 | 0.618 |
| F42A10.5 | 0.647 |
| rpl-12 | 0.649 |
| rpl-41 | 0.655 |
| spd-1 | 0.657 |
| C29F3.7 | 0.659 |
| cyc-1 | 1.511 |
| pat-3 | 1.511 |
| aco-2 | 1.514 |
| T08B2.7 | 1.516 |
| ant-1.1 | 1.526 |
| mup-4 | 1.53 |
| phb-1 | 1.535 |
| F01G4.6 | 1.558 |
| F13C5.5 | 1.565 |
| lys-1 | 1.57 |
| pyc-1 | 1.573 |
| sco-1 | 1.581 |
| C06G3.5 | 1.585 |
| F43G9.1 | 1.587 |
| pccb-1 | 1.605 |
| ttr-45 | 1.636 |
| gex-3 | 1.638 |
| ifa-1 | 1.674 |
| alh-1 | 1.674 |
| ubq-2 | 1.676 |
| ncs-2 | 1.705 |
| T21B6.3 | 1.712 |
| noah-1 | 1.751 |
| C06A6.4 | 1.791 |
| pcca-1 | 1.84 |
| F46G10.1 | 1.843 |
| unc-44 | 1.864 |
| sodh-1 | 1.876 |
| rsd-3 | 1.891 |
| ucr-1 | 1.928 |
| got-2.2 | 1.983 |
| ttr-44 | 2.361 |
| C29E4.13 | 2.53 |
| his-38 | 2.56 |
| his-46 | 2.56 |
| his-31 | 2.56 |
| his-64 | 2.56 |
| his-28 | 2.56 |
| his-18 | 2.56 |
| his-50 | 2.56 |
| his-56 | 2.56 |
| his-26 | 2.56 |
| his-67 | 2.56 |
| his-5 | 2.56 |
| his-37 | 2.56 |
| his-10 | 2.56 |
| his-60 | 2.56 |
| his-1 | 2.56 |
| his-14 | 2.56 |
| srd-59 | 2.941 |
| crh-1 | 4.299 |
