# Supplementary material for: mir-233 Modulates the Unfolded Protein Response in C. elegans during Pseudomonas aeruginosa Infection
Source: PLoS Pathog. 2015 Jan 8;11(1):e1004606. doi: 10.1371/journal.ppat.1004606 (PMC4287614; doi:10.1371/journal.ppat.1004606)
Supplement: S4 Table — The expression of proteins is down-regulated or up-regulated at 8 h post-infection. (DOC) [file ppat.1004606.s018.doc]

**Table S4** **The expression of proteins was down-regulated**

**or up-regulated at 8 h post-infection**

| **Gene name** | **Fold change** |
| --- | --- |
| F15E11.15 | 0.253 |
| rps-24 | 0.278 |
| deb-1 | 0.279 |
| ttr-45 | 0.302 |
| mel-32 | 0.342 |
| ttr-8 | 0.354 |
| rpl-2 | 0.356 |
| icl-1 | 0.374 |
| rpl-43 | 0.387 |
| K07C5.4 | 0.42 |
| K03B8.6 | 0.422 |
| W06A7.2 | 0.45 |
| Y37E3.8 | 0.453 |
| mtl-1 | 0.454 |
| C05C10.3 | 0.46 |
| cey-4 | 0.471 |
| Y39G10AR.8 | 0.475 |
| C37E2.1 | 0.479 |
| T21H3.1 | 0.484 |
| acdh-11 | 0.485 |
| Y37E3.8 | 0.488 |
| Y39G8B.1 | 0.489 |
| inx-12 | 0.493 |
| pgp-1 | 0.494 |
| rpl-11.2 | 0.494 |
| F53A2.7 | 0.506 |
| tufm-1 | 0.52 |
| rps-2 | 0.523 |
| rps-23 | 0.525 |
| his-27 | 0.531 |
| his-17 | 0.531 |
| his-25 | 0.531 |
| his-9 | 0.531 |
| his-2 | 0.531 |
| his-13 | 0.531 |
| his-72 | 0.531 |
| his-49 | 0.531 |
| his-55 | 0.531 |
| his-45 | 0.531 |
| his-42 | 0.531 |
| his-32 | 0.531 |
| his-59 | 0.531 |
| his-71 | 0.531 |
| his-63 | 0.531 |
| his-6 | 0.531 |
| B0334.3 | 0.531 |
| sft-4 | 0.536 |
| sqd-1 | 0.539 |
| mtch-1 | 0.543 |
| T09B4.8 | 0.543 |
| rpl-33 | 0.55 |
| his-66 | 0.551 |
| his-15 | 0.551 |
| his-58 | 0.551 |
| his-52 | 0.551 |
| his-11 | 0.551 |
| his-22 | 0.551 |
| his-4 | 0.551 |
| his-44 | 0.551 |
| his-20 | 0.551 |
| his-54 | 0.551 |
| his-8 | 0.551 |
| his-48 | 0.551 |
| his-29 | 0.551 |
| his-34 | 0.551 |
| his-62 | 0.551 |
| his-41 | 0.551 |
| his-39 | 0.551 |
| rpl-3 | 0.554 |
| F01G4.6 | 0.564 |
| math-33 | 0.565 |
| ant-1.1 | 0.566 |
| dhs-28 | 0.569 |
| C16A3.10 | 0.578 |
| C29F7.2 | 0.578 |
| rps-25 | 0.581 |
| dao-2 | 0.585 |
| cyn-3 | 0.588 |
| rpl-4 | 0.588 |
| unc-60 | 0.59 |
| cyn-13 | 0.59 |
| lap-2 | 0.591 |
| misc-1 | 0.592 |
| Y71F9AL.17 | 0.593 |
| cysl-2 | 0.597 |
| rpl-24.1 | 0.598 |
| spp-5 | 0.6 |
| C23G10.2 | 0.603 |
| rpl-30 | 0.603 |
| C13B9.3 | 0.606 |
| rpt-4 | 0.607 |
| rpt-3 | 0.608 |
| ifb-2 | 0.613 |
| gspd-1 | 0.614 |
| dhs-9 | 0.614 |
| phb-2 | 0.62 |
| lbp-9 | 0.621 |
| F47B10.1 | 0.626 |
| C04C3.3 | 0.626 |
| mcm-4 | 0.626 |
| T22B11.5 | 0.63 |
| ncx-2 | 0.63 |
| rpn-9 | 0.632 |
| sca-1 | 0.635 |
| F45D11.14 | 0.641 |
| rpa-1 | 0.643 |
| rpl-23 | 0.645 |
| rpt-2 | 0.646 |
| eef-1A.2 | 0.648 |
| Y46G5A.4 | 0.648 |
| alh-4 | 0.653 |
| rpl-22 | 0.654 |
| abcf-2 | 0.655 |
| ucr-2.1 | 0.657 |
| alh-13 | 0.666 |
| W03F8.10 | 1.505 |
| phy-2 | 1.506 |
| lfi-1 | 1.507 |
| atp-5 | 1.509 |
| Y38F2AR.9 | 1.517 |
| C14B9.2 | 1.521 |
| ttr-24 | 1.542 |
| cdc-48.2 | 1.563 |
| tag-174 | 1.582 |
| F13E6.1 | 1.609 |
| M28.5 | 1.61 |
| cct-6 | 1.634 |
| pdi-1 | 1.654 |
| gpd-1 | 1.686 |
| gpd-4 | 1.686 |
| myo-1 | 1.726 |
| ttn-1 | 1.727 |
| Y48A6B.3 | 1.762 |
| nuo-4 | 1.765 |
| cal-5 | 1.772 |
| W02D3.12 | 1.776 |
| ncs-2 | 1.789 |
| sax-7 | 1.8 |
| tnc-2 | 1.809 |
| ostd-1 | 1.881 |
| F58F12.1 | 1.908 |
| Y66D12A.9 | 1.933 |
| nucb-1 | 1.945 |
| hrp-2 | 1.952 |
| C16A11.5 | 1.979 |
| rla-0 | 1.986 |
| rsd-3 | 2.071 |
| F42A10.5 | 2.09 |
| prdx-2 | 2.118 |
| F49E2.5 | 2.226 |
| pat-3 | 2.249 |
| gyg-1 | 2.294 |
| T03F1.11 | 2.411 |
| Y105E8B.5 | 2.693 |
| unc-44 | 3.674 |
| gex-3 | 5.661 |
| crh-1 | 6.466 |
| C29E4.13 | 8.054 |
| srd-59 | 14.127 |
| srd-58 | 14.127 |
