# Supplementary material for: mir-233 Modulates the Unfolded Protein Response in C. elegans during Pseudomonas aeruginosa Infection
Source: PLoS Pathog. 2015 Jan 8;11(1):e1004606. doi: 10.1371/journal.ppat.1004606 (PMC4287614; doi:10.1371/journal.ppat.1004606)
Supplement: S5 Table — The expression of proteins is down-regulated or up-regulated at 12 h post-infection. (DOC) [file ppat.1004606.s019.doc]

**Table S5** **The expression of proteins was down-regulated**

**or up-regulated at 12 h post-infection**

| **Gene name** | **Fold change** |
| --- | --- |
| pqn-59 | 0.044 |
| F42A10.5 | 0.111 |
| rps-30 | 0.12 |
| mlc-5 | 0.127 |
| R31.2 | 0.131 |
| far-1 | 0.131 |
| C15C7.5 | 0.133 |
| T28F4.5 | 0.144 |
| hmg-1.1 | 0.151 |
| smo-1 | 0.16 |
| mlc-3 | 0.16 |
| nucb-1 | 0.166 |
| ZC410.5 | 0.169 |
| rps-19 | 0.172 |
| mai-2 | 0.175 |
| gpc-2 | 0.185 |
| tag-174 | 0.185 |
| tag-273 | 0.199 |
| col-41 | 0.211 |
| C42D4.1 | 0.213 |
| R06C1.4 | 0.224 |
| C06A8.3 | 0.235 |
| isp-1 | 0.236 |
| ZK1307.8 | 0.241 |
| R09B3.3 | 0.242 |
| rpl-13 | 0.244 |
| rpl-19 | 0.249 |
| rla-1 | 0.249 |
| grd-5 | 0.257 |
| vha-10 | 0.261 |
| ncs-2 | 0.263 |
| hsp-25 | 0.264 |
| F42G9.1 | 0.267 |
| Y38F2AR.9 | 0.269 |
| pqn-22 | 0.273 |
| calu-1 | 0.274 |
| W02B12.15 | 0.284 |
| eif-3.J | 0.294 |
| Y67H2A.5 | 0.305 |
| npa-1 | 0.305 |
| pfd-1 | 0.314 |
| pqn-94 | 0.322 |
| clec-63 | 0.323 |
| mup-2 | 0.324 |
| ttr-18 | 0.326 |
| ttr-15 | 0.331 |
| emr-1 | 0.332 |
| atp-5 | 0.332 |
| H03A11.2 | 0.339 |
| T28D9.1 | 0.34 |
| snb-1 | 0.34 |
| rps-29 | 0.346 |
| rpl-41 | 0.35 |
| icd-1 | 0.352 |
| F49C12.11 | 0.361 |
| cmd-1 | 0.362 |
| ccg-1 | 0.367 |
| pfd-2 | 0.37 |
| C10G11.7 | 0.38 |
| K03B8.6 | 0.381 |
| rpl-36 | 0.387 |
| grd-14 | 0.389 |
| K06G5.1 | 0.408 |
| enol-1 | 0.41 |
| rmd-2 | 0.411 |
| T20D3.2 | 0.412 |
| nola-3 | 0.415 |
| lea-1 | 0.422 |
| T23E7.2 | 0.423 |
| sax-7 | 0.423 |
| snr-7 | 0.439 |
| C23G10.2 | 0.446 |
| vig-1 | 0.448 |
| ttn-1 | 0.453 |
| W06A7.2 | 0.457 |
| pat-12 | 0.457 |
| tnc-2 | 0.458 |
| rla-0 | 0.458 |
| tbb-2 | 0.462 |
| F45H10.2 | 0.464 |
| F45H10.2 | 0.464 |
| rpl-32 | 0.473 |
| hmg-12 | 0.475 |
| C29F3.7 | 0.497 |
| hsp-17 | 0.502 |
| F49E2.5 | 0.506 |
| hsp-3 | 0.508 |
| phi-44 | 0.509 |
| epi-1 | 0.52 |
| tbb-1 | 0.53 |
| mlc-2 | 0.536 |
| sqd-1 | 0.544 |
| T21B6.3 | 0.553 |
| attf-2 | 0.554 |
| spp-5 | 0.564 |
| cpn-3 | 0.566 |
| gpdh-2 | 0.569 |
| pat-2 | 0.569 |
| F58F12.1 | 0.572 |
| rpl-2 | 0.58 |
| fkb-2 | 0.583 |
| pdi-1 | 0.588 |
| T25C12.3 | 0.59 |
| pat-3 | 0.591 |
| drr-2 | 0.591 |
| asp-1 | 0.6 |
| R10H10.3 | 0.602 |
| mig-6 | 0.602 |
| Y54F10AM.5 | 0.602 |
| mup-4 | 0.607 |
| dim-1 | 0.618 |
| gst-7 | 0.619 |
| mlc-4 | 0.64 |
| his-66 | 0.652 |
| his-58 | 0.652 |
| his-52 | 0.652 |
| his-11 | 0.652 |
| his-22 | 0.652 |
| his-4 | 0.652 |
| his-44 | 0.652 |
| his-20 | 0.652 |
| his-54 | 0.652 |
| his-8 | 0.652 |
| his-48 | 0.652 |
| his-29 | 0.652 |
| his-34 | 0.652 |
| his-62 | 0.652 |
| his-41 | 0.652 |
| his-39 | 0.652 |
| his-15 | 0.652 |
| R04F11.2 | 0.654 |
| rsp-3 | 0.654 |
| T25B6.2 | 0.657 |
| rpl-3 | 1.504 |
| rpt-6 | 1.51 |
| eef-1B | 1.527 |
| Y54E10BR.5 | 1.532 |
| aco-1 | 1.534 |
| rpl-10 | 1.536 |
| unc-44 | 1.552 |
| prl-1 | 1.554 |
| lec-4 | 1.576 |
| K12C11.1 | 1.58 |
| rab-6.2 | 1.606 |
| inx-12 | 1.608 |
| eef-1A.2 | 1.618 |
| eef-1A.1 | 1.618 |
| rps-22 | 1.627 |
| hsp-60 | 1.63 |
| F13C5.5 | 1.635 |
| rack-1 | 1.635 |
| pkg-1 | 1.662 |
| egl-45 | 1.67 |
| cbs-1 | 1.676 |
| gss-1 | 1.728 |
| plst-1 | 1.738 |
| F15C11.2 | 1.739 |
| C06G3.5 | 1.746 |
| K07C5.4 | 1.758 |
| Y24D9A.8 | 1.762 |
| ZK1073.1 | 1.764 |
| pcn-1 | 1.779 |
| rps-25 | 1.798 |
| aos-1 | 1.812 |
| kcc-1 | 1.857 |
| T14G10.5 | 1.871 |
| let-767 | 1.906 |
| phi-30 | 1.916 |
| R05F9.6 | 1.926 |
| pbs-6 | 1.943 |
| idh-2 | 1.946 |
| ran-2 | 1.952 |
| F23B12.5 | 1.956 |
| prmt-1 | 2 |
| rpl-15 | 2.013 |
| C29F7.2 | 2.013 |
| C04C3.3 | 2.034 |
| gcn-1 | 2.035 |
| F14B4.3 | 2.097 |
| rars-1 | 2.101 |
| K10C3.5 | 2.103 |
| dpyd-1 | 2.121 |
| C44B7.10 | 2.125 |
| F38A5.7 | 2.136 |
| abcf-3 | 2.158 |
| clu-1 | 2.171 |
| misc-1 | 2.186 |
| F01G4.6 | 2.216 |
| sco-1 | 2.227 |
| gst-26 | 2.255 |
| gst-27 | 2.255 |
| gst-28 | 2.255 |
| C30C11.4 | 2.273 |
| imb-3 | 2.296 |
| F47B10.1 | 2.329 |
| rps-23 | 2.335 |
| nuo-1 | 2.341 |
| alh-13 | 2.359 |
| pmp-4 | 2.374 |
| acdh-3 | 2.38 |
| cct-6 | 2.445 |
| dhc-1 | 2.448 |
| noah-1 | 2.451 |
| pyr-1 | 2.554 |
| ctl-2 | 2.56 |
| rps-7 | 2.591 |
| B0334.3 | 2.868 |
| Y71F9AL.17 | 3.008 |
| czw-1 | 3.206 |
| abcf-2 | 3.271 |
| T22B11.5 | 3.326 |
| dhs-28 | 3.694 |
| E01A2.1 | 3.893 |
| dao-3 | 3.907 |
| fasn-1 | 4.359 |
| C29E4.13 | 14.054 |
| crh-1 | 23.283 |
| gex-3 | 30.536 |
