# Supplementary material for: mir-233 Modulates the Unfolded Protein Response in C. elegans during Pseudomonas aeruginosa Infection
Source: PLoS Pathog. 2015 Jan 8;11(1):e1004606. doi: 10.1371/journal.ppat.1004606 (PMC4287614; doi:10.1371/journal.ppat.1004606)
Supplement: S6 Table — The miRNA mutants in this study. (DOC) [file ppat.1004606.s020.doc]

**Table S6 The miRNAs mutants in this study**

| mir-233(n4761) | mir-83(n4638) | mir-52(n4100) | mir-239(ndf62) |
| --- | --- | --- | --- |
| mir-232(ndf56) | mir-87(n4104) | mir-124(n4255) | mir-48, mir-241(ndf51) |
| mir-67(n4899) | mir-45(n4280) | mir-1(n4101) | mir-81-82(ndf54) |
| mir-86(n4607) | mir-53(n4113) | mir-235(n4504) | mir-54-56(ndf58) |
| mir-85(n4117) | mir-51(n4473) | mir-75(4471) | mir-64,mir-229(ndf52) |
| mir-63(n4568) | mir-71(n4115) | mir-77(n4286) | mir-64-66,mir-229(ndf63) |
| mir-243(n4759) | mir-72(n4130) | mir-2(n4108) | mir-73-74(ndf47) |
| mir-84(n4037) | mir-1(n4102) | mir-59(n4604) | mir-247, mir-797(n4505) |
| mir-245(n4798) | let-7(n2853) | mir-46(n4475) | mir-42-44(ndf49) |
| mir-62(n4539) | mir-58(4640) | mir-47(gk167) | mir-61, mir-250(ndf59) |
| mir-259(n4106) | lys-6(ot71) | mir-60(n4947) | mir-240,mir-786(n4541) |
| mir-34(n4276) | mir-79(n4126) | mir-238(n4112) |  |
